# Polaron interfacial entropy as a route to high thermoelectric performance in DAE-doped PEDOT:PSS films

**DOI:** 10.1093/nsr/nwae009

**Published:** 2024-01-09

**Authors:** Jiajia Zhang, Caichao Ye, Genwang Wei, Liang Guo, Yuhang Cai, Zhi Li, Xinzhi Wu, Fangyi Sun, Qikai Li, Yupeng Wang, Huan Li, Yuchen Li, Shuaihua Wang, Wei Xu, Xuefeng Guo, Wenqing Zhang, Weishu Liu

**Affiliations:** Department of Materials Science and Engineering, Southern University of Science and Technology, Shenzhen 518055, China; Department of Materials Science and Engineering, Southern University of Science and Technology, Shenzhen 518055, China; Academy for Advanced Interdisciplinary Studies and Guangdong Provincial Key Laboratory of Computational Science and Material Design, Southern University of Science and Technology, Shenzhen 518055, China; Department of Materials Science and Engineering, Southern University of Science and Technology, Shenzhen 518055, China; Academy for Advanced Interdisciplinary Studies and Guangdong Provincial Key Laboratory of Computational Science and Material Design, Southern University of Science and Technology, Shenzhen 518055, China; Department of Mechanical and Energy Engineering, Southern University of Science and Technology, Shenzhen 518055, China; Department of Mechanical and Energy Engineering, Southern University of Science and Technology, Shenzhen 518055, China; Department of Mechanical and Energy Engineering, Southern University of Science and Technology, Shenzhen 518055, China; Department of Materials Science and Engineering, Southern University of Science and Technology, Shenzhen 518055, China; Department of Materials Science and Engineering, Southern University of Science and Technology, Shenzhen 518055, China; Department of Materials Science and Engineering, Southern University of Science and Technology, Shenzhen 518055, China; Department of Materials Science and Engineering, Southern University of Science and Technology, Shenzhen 518055, China; Department of Materials Science and Engineering, Southern University of Science and Technology, Shenzhen 518055, China; Department of Materials Science and Engineering, Southern University of Science and Technology, Shenzhen 518055, China; Department of Materials Science and Engineering, Southern University of Science and Technology, Shenzhen 518055, China; Beijing National Laboratory for Molecular Sciences, Key Laboratory of Organic Solids, Institute of Chemistry, Chinese Academy of Sciences, Beijing 100190,China; College of Chemistry and Molecular Engineering, National Biomedical Imaging Centre, Peking University, Beijing 100091, China; Department of Materials Science and Engineering, Southern University of Science and Technology, Shenzhen 518055, China; Academy for Advanced Interdisciplinary Studies and Guangdong Provincial Key Laboratory of Computational Science and Material Design, Southern University of Science and Technology, Shenzhen 518055, China; Department of Materials Science and Engineering, Southern University of Science and Technology, Shenzhen 518055, China; Guangdong Provincial Key Laboratory of Functional Oxide Materials and Devices, Southern University of Science and Technology, Shenzhen 518055, China

**Keywords:** thermoelectric, PEDOT:PSS, interfacial occupied entropy, resonant coupling, UV-light modulation

## Abstract

Enhancing the thermoelectric transport properties of conductive polymer materials has been a long-term challenge, in spite of the success seen with molecular doping strategies. However, the strong coupling between the thermopower and the electrical conductivity limits thermoelectric performance. Here, we use polaron interfacial occupied entropy engineering to break through this intercoupling for a PEDOT:PSS (poly(3,4-ethylenedioxythiophene)-poly(4-styrenesulfonate)) thin film by using photochromic diarylethene (DAE) dopants coupled with UV-light modulation. With a 10-fold enhancement of the thermopower from 13.5 μV K^−1^ to 135.4 μV K^−1^ and almost unchanged electrical conductivity, the DAE-doped PEDOT:PSS thin film achieved an extremely high power factor of 521.28 μW m^−1^ K^−2^ from an original value of 6.78 μW m^−1^ K^−2^. The thermopower was positively correlated with the UV-light intensity but decreased with increasing temperature, indicating resonant coupling between the planar closed DAE molecule and PEDOT. Both the experiments and theoretical calculations consistently confirmed the formation of an interface state due to this resonant coupling. Interfacial entropy engineering of polarons could play a critical role in enhancing the thermoelectric performance of the organic film.

## Teaser

By directly manipulating the interfacial polaron occupation entropy in a PEDOT:PSS-DAE thermoelectric thin film through UV-light illumination, a 10-fold increase in thermopower has been achieved without affecting conductivity.

## INTRODUCTION

Conductive polymers have been studied extensively for thermoelectric power generation due to their flexibility [1–8]. The Seebeck coefficient (or thermodiffusion thermopower) represents the transport entropy of the unit charge carriers [9–11]. In conductive polymers, the charge carrier is referred to as a polaron or bipolaron, i.e. electrons or holes that are partially localized due to electron–phonon coupling [12]. However, compared to inorganic thermoelectric materials, the low Seebeck coefficient is one of the major shortcomings limiting the total thermoelectric performance; it is scaled by the dimensionless figure of merit *ZT*, which is defined as *ZT* = *S^2^σ*/*κ*, where *S, σ, T* and *κ* are the Seebeck coefficient (or thermopower), electrical conductivity, absolute temperature and thermal conductivity, respectively [13].

Among organic thermoelectric materials, PEDOT:PSS (poly(3,4-ethylenedioxythiophene)-poly(4-styrenesulfonate)) thin films have received a lot of attention but suffer from low thermopowers of 10–20 μV K^−1^ due to the high oxidation states in the PEDOT polymer chain [14–17]. Considerable effort has been devoted to increasing the thermopower. One strategy is to alter the oxidation state of the ethylenedioxythiophene unit from quinoid to benzoid by post-treatment with a reducing solution. Li *et al.* [18] reported enhanced thermopowers of PEDOT:PSS films ranging from 14 to 21.9 μV K^−1^ near room temperature after they were subject to L-ascorbic acid (LAA) immersion post-treatments, indicating an optimized power factor up to 114 μW m^−1^ K^−2^ at 150°C. Yemata *et al.* achieved a similar optimization with sodium formaldehyde sulfoxylate [19] or hydrazine post-treatments [20], which provided an optimized thermopower up to 51.8 μV K^−1^ and a power factor of 185 μW m^−1^ K^−2^. Note that a high thermopower over 100 μV K^−1^ is accessible by using a strong reductant, such as NaBH_4_, but there is also a three-orders-of-magnitude decrease in electrical conductivity because the system is far from having the optimized charge carrier concentration [21]. Therefore, it is still a challenge to increase the thermopower without decreasing the electrical conductivity due to the intrinsic coupling. Alternatively, enhancement of thermoelectric performance was achieved by adding inorganic thermoelectric nanoparticles such as Bi_2_Te_3_ and Te [22–26]. Cai *et al.* [27] reported simultaneous increases in both the thermopower and the electrical conductivity in a PEDOT:PSS/Bi_2_Te_3_ composite film, which showed an optimized thermopower of 21 μV K^−1^ and a power factor of 32.6 μW m^−1^ K^−2^ with 4.1 wt% Bi_2_Te_3_ nanosheets. A high thermopower of 163 μV K^−1^ was observed for a PEDOT:PSS/Te composite film with 85 wt% Te nanorods [22]. The very high thermopower might have resulted from the connected Te network. Some oxide nanoparticles, such as BaTiO_3_ [28], were also reported to increase the thermopower. In contrast to the commercially available PEDOT:PSS, Crispin *et al.* [6] synthesized a PEDOT:Tos film by *in situ* polymerization of EDOT monomers and iron(ш) tris-p-toluenesulfonate, which showed a high thermopower of 55 μV K^−1^ and a high electrical conductivity of 1500 S cm^−1^ because it was more crystalline than PEDOT:PSS. However, it remains difficult to overcome the intrinsic coupling between the thermopower and electrical conductivity.

Here, we report direct manipulation of the polaron interfacial occupied entropy in a PEDOT:PSS thin film with UV-light-induced resonance between PEDOT and diarylethene, thereby realizing a 10-fold thermopower enhancement from 13.5 to 135.4 μV K^−1^ with almost unchanged electrical conductivity. The closed-ring diarylethene and PEDOT share similar C−C=C−C carbon skeletons and form a new interface that increases the interfacial entropy of the polarons and increases the thermopower. Our work provides a new strategy for decoupling the thermopower and electrical conductivity of organic thermoelectric materials with typical polaron transport and provides insight into the enhancement of thermoelectric performance.

## RESULTS AND DISCUSSION

### Polaron interfacial occupied entropy ($\hat{S}$*_p-__interf_*) engineering

We introduced an interface state to tailor the polaron interfacial occupied entropy of PEDOT:PSS with a photochromic diarylethene (DAE). At an interface between two materials, this interaction can result in the formation of polaron states associated with the charge carriers from each material. A polaron interfacial state is a unique electronic state that emerges at the boundary or interface between two materials. It is characterized by the presence of polarons in this specific region, and it may exhibit distinct electronic and transport properties compared to the bulk of either material. The formation of such states is influenced by the interactions between the lattice distortions (polarons) and the materials involved. Polaron interfacial states are of interest in various fields, including condensed matter physics and materials science, because they can have a significant impact on the electronic and optoelectronic properties of interfaces. These states may contribute to the modification of charge transport and energy band alignments.

The term ‘interfacial occupied entropy’ specifically relates to the entropy associated with the occupation or presence of these polarons at the interface. The polarons effectively introduce new electronic states or sites where charge carriers can be accommodated. The occupation of these states contributes to increased entropy because it expands the possible arrangements of charge carriers. This increase in entropy can have a significant impact on the thermopower (Seebeck coefficient) of the material. The thermopower measures a material's ability to generate an electrical voltage in response to a temperature gradient. More available electronic states, as provided by the polarons at the interface, can result in an increase in the thermopower by allowing more charge carriers to participate in the thermoelectric process. In summary, ‘polaron interfacial occupied entropy’ represents the increased disorder and randomness in the electronic states at the interface of two materials due to the presence of polarons. This increased entropy can enhance the thermoelectric properties, specifically the thermopower, by providing more opportunities for charge carriers to contribute to thermoelectric voltage generation.

**Figure 1.**
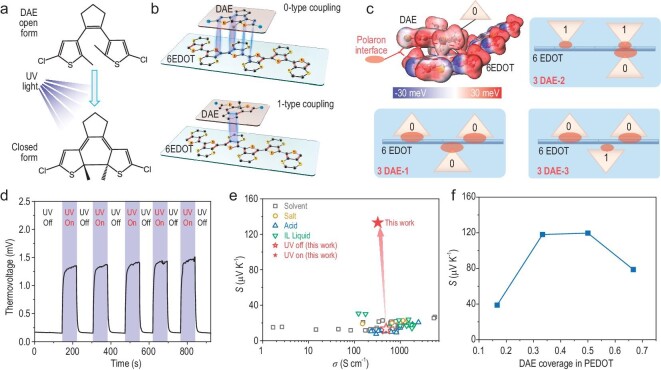
Polaron interfacial occupied entropy ($\hat{S}$*_p-__interf_*) engineering. (a) Photochromic reaction scheme for DAE between the open and closed molecular conformations under UV irradiation. (b) Schematic showing the different coupling states for DAE and 6EDOT. (‘0-type coupling’ indicates that the C−C=C−C cyclopentene bonds of DAE are oriented in the same direction as the carbon skeleton of the PEDOT polymer. ‘1-type coupling’ indicates that the C−C=C−C cyclopentene bonds of DAE are oriented in the opposite direction from the carbon skeleton of the PEDOT polymer.) (c) Electrostatic potential of the *0*-type coupling state 6EDOT-1DAE and schematic of the 6EDOT-3DAE triply degenerate states with different coupling forms. The red ellipse represents the polaron interface states. (d) Thermovoltage between hot and cold end evolution of a PEDOT:PSS-*x*DAE (*x* = 38 wt%) sample with the UV light turned on or off (the UV laser power was 1.3 W cm^−2^). (e) The thermopower-electrical conductivity relationships of PEDOT:PSS films made with different treatment methods [16,17,52–59]. (f) The increasing thermopower resulting from a polaron interfacial occupied entropy calculation for DAE molecules and six EDOT units with different degeneracies.

Figure 1a shows a schematic describing the stereo structural transformations of DAE molecules from open-ring structure to closed-ring form, under UV light [29]. A new polaron interface state was formed between the planar closed-ring DAE and the PEDOT molecular chains due to their similar C−C=C−C bonds, which were coupled with each other via weak interactions. More detailed experimental and theoretical evidence will be presented later. Based on the coupling direction, we defined *0*-type coupling with the same direction between the two C−C=C−C coupled bonds and *1*-type coupling with opposite directions as the two molecules come together (Fig. 1b). Singlet *1*-type coupling has a slightly higher energy, ∼15.2 meV, than *0*-type coupling, but the difference is much less than 1 *k_B_T* (∼23.55 meV near room temperature). This minor energy difference added to the configurational complexity of the interfacial state for a specific proportion of the DAE-doped PEDOT systems. Here, we considered a degenerate polaron interfacial state because the Gibbs coupling energy difference among different configurational complexes was <1 *k_B_T* and the polaron interfacial state was at the same energy level (highest occupied molecular orbital (HOMO) for p-type material here, <1 *k_B_T*). The electrostatic potential of the *0*-type coupling state of 6EDOT-1DAE is shown in Fig. 1c to demonstrate the polaron interface state (positive polaron for p-type material here). In the case of 3DAE on 6EDOT, there were 9 configurations, while the polaron interfacial state degeneracy was 3 according to the definition for a difference <1 *k_B_T*, as shown in Fig. 1c. The detailed Gibbs coupling energy data and polaron interfacial state energy level are shown in Figs S1–S4 and Tables S1–S4. Notably, the disordered Co^3+^ and Co^4+^ valence states in Na*_x_*Co_2_O_4_ provided a rich electronic state to increase the electronic entropy, hence the thermopower [30,31]. Similarly, the disordered *0*-type and *1*-type coupling for polaron states at the DAE and PEDOT interface added numerous new polaron occupancy sites and increased the polaron interfacial entropy, which could have a positive impact on the thermopower. Later, we will discuss how the polaron interface states increased the thermopower.

We designed a series of DAE-doped PEDOT:PSS thin films (thickness: 100–200 nm) with the general formula PEDOT:PSS-*x*DAE (*x* = 16, 28, 38, 44 and 50% in weight ratio). Figure 1d shows the transient thermovoltage measurement of the as-fabricated PEDOT:PSS-*x*DAE (*x* = 38 wt%) with a fixed temperature difference of Δ*T* = 11 K and on-off cycling of UV light with a wavelength of 355 nm and power density of 1.3 W cm^−2^. In the UV off state, the PEDOT:PSS-38wt%DAE thin film (thickness: 150 nm) displayed a 0.15 mV potential indicating a typical low thermopower of ∼13 μV K^−1^, which was consistent with previous reports [16–18,32,33]. However, under UV irradiation, the thermovoltage increased rapidly from 0.15 mV to 1.43 mV within 30 s and then became saturated. When the UV light was turned off, the thermovoltage dropped back to 0.15 mV within 30 s. The closed-ring DAE molecules have higher energies than the open-ring form [34], so they returned to the low-energy open-ring form when the UV light was off. The UV on/off-dependent thermovoltages were highly repeatable (shown in Fig. S8), consistent with the intrinsic dynamic conformational transitions of the DAE molecules upon introduction of UV light [29]. We have also used 365 nm and 395 nm UV light to investigate the thermoelectric properties of the PEDOT:PSS-*x*DAE film, as shown in Fig. S10 and Fig. S11. Our findings indicate that the 365 nm UV light had a noticeable impact, resulting in a significant enhancement of thermopower (from 11.69 to 39.51 μV K^−1^) for the PEDOT:PSS-38wt%DAE film. In contrast, the 395 nm UV light did not yield substantial improvements in thermopower (from 13.54 to 15.27 μV K^−1^) for the PEDOT:PSS-38wt%DAE film. We postulate that the relatively low-energy 395 nm UV light may not effectively trigger the transformation of open-form DAE molecules into their closed form. This lack of transformation does not lead to the production of polaron interfacial states between the DAE molecules and the PEDOT chain. Note that the measured samples were placed in the center of the beam to ensure synchronous temperature changes of the hot and cold electrodes, while the hot and cold gold electrodes were covered with a light-block tap to prevent an electron injection effect from the electrode. The previous study [35] has confirmed that the electrode/polymer interface could affect the total thermopower due to entropy difference within the internal energy of charge carriers when injected between high-temperature and low-temperature metal electrodes. Importantly, when the temperature gradient was 0 between the hot electrode and cold electrode, we observed that there was no photo-voltage (Fig. S9) generated, whether UV light was switched off or on. Furthermore, the thermopower of the PEDOT:PSS-*x*DAE thin film under UV light was measured with the steady method according to the formula *S= −* (*V_H_ − V_C_*)/(*T_H_ − T_C_*), where *V*_H_—*V*_C_ is the voltage difference and *T*_H_ − *T*_C_ is the temperature difference (Figs S6–S11). It was shown that a high thermopower of 132.4 μV K^−1^ was obtained depending on the UV-light modulation, and this corresponded to a 10-fold increase on the value at the intrinsic state (UV light off) with a negligible impact on electrical conductivity (Fig. 1e). The extremely high thermopower was related to the UV-light-modulated interfacial configurational occupied entropy increase since the formation of the planar form of the closed-ring DAE is highly dependent on the UV light. When the UV light was turned off, the open-ring DAE molecule adopted a stereo structure that could not fit the C−C=C−C bonds in the PEDOT chains and the photoexcitation ceased, so the polaron interfacial state was lost and the system reverted to its original state.

According to statistical thermodynamics, entropy ($\hat{S})$ is a logarithmic measure of the number of system states with a significant occupation probability [36]:


\begin{eqnarray*}
\hat{S} = - {k}_B\mathop \sum \limits_i {p}_i{\rm ln}{p}_i
\end{eqnarray*}

where ${p}_i$ is the probability that the system is in the *i*th state and ${k}_B$ is the Boltzmann constant. For organic thermoelectric materials, the flow of charge carriers (polarons) along the temperature gradient $- \nabla T$ leads to an entropy change that determines the thermopower ($S = \hat{S} / e$) [24]. The polaron transport entropy is associated with the disorder or randomness of the hopping processes. It represents the degree of uncertainty or the multiplicity of states available for occupation by the charge carriers during transport. Coupled small molecule doping with conducting polymers could form new polaron interfacial states between the polymer chains and the small molecules, which would provide extra polaron-occupied sites at the interface. The presence of these sites would modify the energy levels and increase the density of states (Fig. S5). A higher density of available energy levels enables more efficient energy conversion and transport processes, leading to a larger thermopower. It is important to clarify the relationship between the polaron interfacial occupied entropy and the thermopower.

Since the DAE dopant content is a variable, the increased thermopower (${S}_{\mathit{inter\! f}}$) caused by the polaron interfacial occupied entropy of the DAE molecules and PEDOT chain system is calculated with the following formula:


(1) \begin{eqnarray*}
{S}_{\scriptstyle{ inter\!f}} = - \frac{{{k}_B}}{e}\left( {n \cdot {\rm ylny} + \left( {1 - y} \right)\ln\left( {1 - y} \right)} \right)
\end{eqnarray*}

where *y* is the coverage of DAE molecules in the PEDOT chains and *n* is the degeneracy (the formula deduction and degeneracy definition are shown in Supplementary sections 3 and 4). The PEDOT:PSS-*x*DAE (*x* = 38 wt%) system, which is similar to the 6EDOT-4DAE system, had the highest coupling concentration. According to our calculated results (Figs S1–S4), the degeneracies of 6EDOT-1DAE, 6EDOT-2DAE, 6EDOT-3DAE and 6EDOT-4DAE are 1, 3, 3 and 2, respectively. The theoretical thermopower, which is based on the polaron interfacial occupied entropy, predicts increasing thermopower with increases in the DAE concentration, with a maximum thermopower near the case for 6EDOT-3DAE, and then a decrease when *m* > 3. It was shown that the experimental value of ∼132.4 μV K^−1^ of PEDOT:PSS-*x*DAE (*x* = 38 wt%) was very close to the theoretical value (∼119.56 μV K^−1^, shown in Fig. 1f) for the case 6EDOT-3DAE with a degeneracy *n* = 3 plus the intrinsic thermopower (∼12 μV K^−1^). The strikingly high thermopower of the UV-light-modulated PEDOT:PSS-*x*DAE films shares physical similarities with the Na*_x_*Co_2_O_4_ system [30,31] but provides extra dimensions with which to manipulate the polaron interfacial occupied entropy.

### Optimization of the thermopower

**Figure 2.**
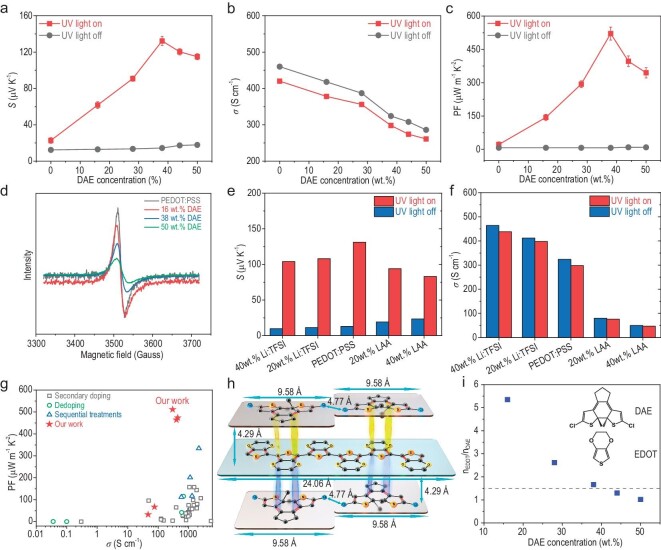
Optimization of the thermopower. (a–c) Comparison of the thermopower, electrical conductivity and power factor of PEDOT:PSS-*x*DAE films versus the DAE concentration with the UV light off and with the UV light on (UV power = 1.3 W cm^−2^). (d) EPR signals for PEDOT:PSS-*x*DAE films with various DAE concentrations. (e–f) The thermopower and electrical conductivity of the PEDOT:PSS-*x*DAE (*x* = 38 wt%) sample with the UV light off and with the UV light on (UV power = 1.3 W cm^−2^) after doping with the ionic liquid Li : TFSI and de-doping with L-ascorbic acid. (g) Power factor versus electrical conductivity plot for PEDOT:PSS films made with different treatment methods [16,17,18,32,33,53,55,60–64]. (h) The closest arrangement of DAE molecules with 6 EDOT (3,4-ethylenedioxythiophene) units. (i) Molar ratio of EDOT and DAE units as the DAE concentration was increased.

Figure 2a–c shows the effect of DAE concentration on the thermoelectric transport properties of the as-prepared PEDOT:PSS-*x*DAE thin films with the UV light on and off. Firstly, with the UV light off, the thermopower increased slightly from 12.4 to 17.9 μV K^−1^, while the electrical conductivity decreased from 460 to 286 S cm^−1^ as the DAE content was increased from *x* = 0 wt% to 50 wt% (Fig. 2a and b). Specifically, the PEDOT chains in their neutral, polaron and bipolaron states show absorption at ≈600, ≈900 and ≈1400 nm, respectively [37–42]. According to the UV-Vis spectra in Fig. S20a, both the polaron and bipolaron concentrations of PEDOT:PSS-*x*DAE films decrease with increasing DAE content. The polaron and bipolaron contents have almost no changes before and after UV-light conditions, as is shown in Fig. S20b. We also measure the electrical conductivity of pure DAE molecules (Fig. S17a), and it is <6 × 10^−13^ S cm^−1^ under UV light off and 2 × 10^−12^ S cm^−1^under UV light on, which is about 14–15 orders of magnitude lower than the PEDOT:PSS film. The DAE molecules are almost insulating due to the ultralow charge carrier concentration, and we think that the decrease in electrical conductivity is not the de-doping effect. The decrease in electrical conductivity resulted from the addition of DAE introducing structural disruptions and lowering the conjugation along the polymer chain, which hindered the efficient delocalization of charges and reduced the electrical conductivity of the PEDOT:PSS materials. This mainly decreases the charge carrier's mobility. With the UV light on (light intensity = 1.3 W cm^−2^, incidence angle = 0^o^), the thermopower rapidly increased from 19.4 to a peak value of ∼135.5 μV K^−1^ as the DAE content was increased from *x* = 0 wt% to *x* = 38 wt%, and then decreases slightly to ∼114.9 μV K^−1^ at *x* = 50 wt% due to the content of DAE being over the maximum coupling level, i.e. 6EDOT-4DAE corresponding to ∼*x* = 38 wt%. Too many DAE molecules might decrease the coupling between the DAE and PEDOT due to the steric effect. Compared with the 10-fold enhancement in the thermopower, the UV-light-on electrical conductivity was only ∼10% lower than the UV-light-off values for all the investigated PEDOT:PSS-*x*DAE thin films. The ultra-low charge carrier concentration in pure DAE films leads to a nearly negligible thermoelectric effect. It suggested the interfacial states capturing charge carriers and slightly preventing their free movement through the PEDOT:PSS matrix. In addition, it is widely accepted that the thermopower of PEDOT:PSS is directly defined by the slope of the density of electronic states near *E_F_* [43,44]. We also calculate the density of electronic states at varying concentrations of closed-ring DAE versus 6EDOT, as illustrated in Fig. S5a and b. Remarkably, with the addition of closed-ring DAE, the slope of the density of electronic states near *E_F_* increased compared with the pure PEDOT, which is also consistent with the observed trend of enhanced thermopower.

As a result, an optimized power factor of 521 μW m^−1^ K^−2^ was reached with the PEDOT:PSS-38wt%DAE thin film with the UV light on, which was 93 times higher than the value seen with the UV light off (Fig. 2c). The Electron Paramagnetic Resonance (EPR) signals (Fig. 2d) represent the polaron concentration in the PEDOT:PSS-*x*DAE film, which is consistent with the absorption peak at ∼900 nm in UV-Vis spectra (Fig. S20a). It explains the plot of electrical conductivity decreased tendency versus DAE concentration increase. It was clear that UV-light-modulating polaron interfacial occupied entropy engineering decoupled the connection between the thermopower and the electrical conductivity. The light-modulation-enhanced thermopower in this work is very different from that seen in previous studies, which mainly came from an electrode effect, such as in the studies of Hu *et al.* [35] on an Al/PEDOT:PSS/Al device, Xu *et al.* [45] on ITO (hot)/MEH-PPV/Au (cold) and Zhu *et al.* [46] on Au/poly[Cux(Cu-ett)] : PVDF/Au thin films; the changes in the Density of States (DOS) distribution: a larger DOS distribution indicating the increased energetic disorder and thermopower, e.g. Zhu *et al.* [47] with NDI(2OD) (4tBuPh)-DTYM2; and the de-doping effect, e.g. Ouyang *et al.* [48,49] with PEDOT:PSS and TiO_2_ nanoparticles or two-dimensional potassium poly-(heptazine imide) (KPHI).

Figure 2e–g shows a comparison of the UV-light-modulation effect on the thermoelectric power of the various PEDOT:PSS-38wt%DAE thin films with different charge carrier concentrations formed by changing the oxidation states. Here, we utilized Li:TFSI (Bis(trifluoromethane)sulfonimide lithium salt) as the oxidant to increase the intrinsic carrier concentration, and LAA as the reductant to decrease the carrier concentration. With increases in the carrier concentration, the UV-light-off thermopower of the PEDOT:PSS-38wt%DAE-y Li : TFSI system decreased from 13 to 11.4 and 9.86 μV K^−1^, while the electrical conductivity increased from 324 to 398 and 438 S cm^−1^ as the Li : TFSI content increased from y = 0 wt% to 20 wt% and 40 wt%. In contrast, the UV-light-off thermopower of the PEDOT:PSS-38wt%DAE-z LAA light system increased from 13 to 19.2 and 23.4 μV K^−1^, while the electrical conductivity decreased from 298 to 76 and 47 S cm^−1^ as the content of LAA increased from z = 0 wt% to 20 wt% and 40 wt%. It was also verified that decreasing the charge carrier concentration only slightly increased the thermopower but with a high electrical conductivity decrease. The UV-light-on thermopowers for both the PEDOT:PSS-38wt%DAE-y Li : TFSI and PEDOT:PSS-38wt%DAE-z LAA thin films consolidated the thermopower enhancement effect of polaron interfacial occupied entropy engineering. Note that the addition of Li : TFSI and LAA decreased the thermopower enhancement ratios. The steric effects of the dopant molecule impacted the coupling between the DAE and PEDOT and finally changed the polaron interfacial occupied entropy of the charge carrier. Figure 2g compares the power factors of the as-fabricated PEDOT:PSS-38wt%DAE, PEDOT:PSS-38wt%DAE-y Li : TFSI and PEDOT:PSS-38wt%DAE thin films, together with those for the other reported PEDOT:PSS thin films. The advantage of the polaron-interfacial-occupied-entropy-engineered PEDOT:PSS thin film is clearly shown.

Figure 2h and i shows the molar ratio of the EDOT units and the DAE molecules in the as-fabricated PEDOT:PSS-*x*DAE thin film. The optimized PEDOT:PSS-38wt%DAE showed a ratio of 1 : 1.68, which was very close to the case for 4DAE coupled with 6EDOT in a ratio of 1 : 1.5. In theory, the highest thermopower is anticipated for the 6EDOT-3DAE compound. However, in practical experiments, the highest thermopower is achieved with PEDOT:PSS containing 38 wt% DAE. This outcome aligns with the composition of 6EDOT-4DAE, which exhibits the most favorable coupling concentration. When introducing DAE molecules into PEDOT:PSS, there is a possibility that some DAE molecules cannot effectively bond with the PEDOT chain due to steric hindrance effects arising from the mutual repulsion of the two chlorine atoms in DAE molecules. Consequently, in experimental settings, a slightly higher quantity of DAE molecules may be necessary for PEDOT to reach the maximum achievable thermopower value. Figure 2h presents the steric configuration, which suggests that the 6EDOT-4DAE configuration represents the maximum coupling number. More DAE molecules would have a detrimental steric effect on the coupling between the DAE and PEDOT, as seen with the extra Li : TSFI and LAA dopants.

### Resonant coupling between DAE and PEDOT

**Figure 3.**
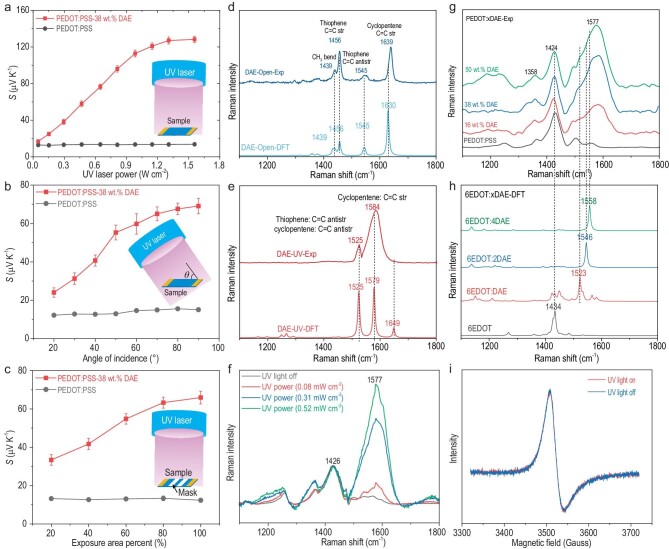
Resonant coupling between DAE and PEDOT. (a–c) UV-light modulation increased the thermopower. Thermopower of PEDOT:PSS-38wt%DAE and PEDOT:PSS as a function of UV laser power, angle of incidence light (UV power = 0.47 W cm^−2^) and exposure area percentage (UV power = 0.47 W cm^−2^). (d) Experimental and calculated Raman spectra for DAE with the UV light off. (e) Experimental and calculated Raman spectra for DAE with the UV light on. (f) Raman spectra of PEDOT:PSS-38wt%DAE films with different UV power densities. (g) Experimental Raman spectra of PEDOT:PSS and PEDOT:PSS-*x*DAE (*x* = 16 wt%, 38 wt% and 50 wt%) films with the UV light on. (h) Calculated Raman spectra for PEDOT:PSS and PEDOT:PSS-*x*DAE (*x* = 16 wt%, 38 wt% and 50 wt%) films with the UV light on. (i) EPR signals of PEDOT:PSS-38wt%DAE films with the UV light off and with the UV light on.

Systematic experiments were carried out to verify the resonant coupling between DAE and PEDOT. Figure 3a–c presents the effect of UV-light intensity on the thermopower of the optimized PEDOT:PSS-38wt%DAE and pure PEDOT:PSS films. Firstly, we investigated the effect of laser power density on the thermopower by tuning the pulse energy of the 355 nm UV laser (Fig. 3a). We observed an improvement in the thermopower of the PEDOT:PSS-38wt%DAE film from 17.5 to 132.0 μV K^−1^ as the laser power density was increased from 0.05 W cm^−2^ to 1.3 W cm^−2^, and then the thermopower exhibited a saturated state with the UV laser density that was increased to 1.56 W cm^−2^ further. We believe that the phenomenon is attributable to the saturation of closed-ring DAE molecules. In contrast, the thermopower of the pure PEDOT:PSS film showed a slight change from 14.8 to 18.9 μV K^−1^, which could be a purely thermal effect. Next, we used a fixed UV laser power density of 0.47 W cm^−2^ and investigated the impacts of the UV-light angle of incidence (Fig. 3b) and the exposure area (Fig. 3c) on the thermopower of the as-fabricated PEDOT:PSS-DAE thin film. The thermopower of the PEDOT:PSS-38wt%DAE film decreased from ∼69.1 to ∼24.0 μV K^−1^ as the UV source angle of incidence was increased from 0° to 90°, but the thermopower of the pure PEDOT:PSS decreased slightly from ∼15.51 to ∼12.18 μV K^−1^. Then, we partially covered the samples with aluminium foil. When we adjusted the exposure area from 20% to 100%, the thermopower of the PEDOT:PSS-38wt%DAE film increased from ∼33.4 to ∼65.9 μV K^−1^. In contrast, the thermopower of the pure PEDOT:PSS film was almost maintained at ∼13 μV K^−1^. Both the angle of incidence for the UV light and the exposure area changes could affect the actual UV-light intensity directed on the PEDOT:PSS-*x*DAE film. As the UV laser power density increases, there is a proportional increase in the number of photons engaged in driving the closed reaction of the DAE molecule, resulting in a greater number of high-energy closed-ring DAE molecules forming the interface state with the PEDOT chains. This, in turn, contributes to increasing the polaron interfacial occupied entropy within the PEDOT:PSS-*x*DAE film, leading to an enhancement in the thermopower of the material.

We also used Raman spectra to seek direct experimental evidence for the coupling between DAE and PEDOT under UV modulation. First, the open-ring DAE molecule has two strong Raman scattering peaks at ∼1456 cm^−1^ and ∼1639 cm^−1^, which correspond to the symmetric stretching vibrations of the C=C bonds of the thiophene units and cyclopentene, and two relatively weak peaks at ∼1545 cm^−1^ (antisymmetric C=C stretching vibration) and 1439 cm^−1^ (–CH_3_ bending modes) (Fig. 3d). In contrast, the closed-ring DAE showed a peak at 1584 cm^−1^ resulting from stretching vibrations of two in-plane C=C bonds (Fig. 3e). The experimentally observed Raman spectra were consistent with the spectrum calculated theoretically with B3LYP at the 6–31G(d) level with the Gauss 16 package [50]. Figure 3f shows the power density of the UV light irradiated on the as-fabricated PEDOT:PSS-38wt%DAE film to determine the Raman spectrum. The intensity of the broad Raman peak near 1577 cm^−1^ was strongly connected with the UV-light power density, which showed that there was more closed-ring DAE formation. It is consistent with the thermopower increasing tendency versus UV-light power density in Fig. 3a.

Figure 3g shows the Raman spectrum of the as-fabricated PEDOT:PSS-*x*DAE thin film with the UV light on. The prominent peak at 1424 cm^−1^ indicated symmetric C=C stretching vibrations in the PEDOT chains, while the other weak peaks at 1364, 1506 and 1543 cm^−1^ were attributed to symmetric C−C stretching vibrations, antisymmetric C=C stretching vibration of the thiophene units in the PEDOT chains, and a symmetric quinoid C=C stretching vibration, respectively. A broad Raman scattering peak was observed near ∼1577 cm^−1^, which differed from the narrow peak at 1584 cm^−1^ for the pure closed-ring DAE. This broad Raman peak was enhanced with increasing DAE concentrations. Figure 3h shows the theoretical Raman spectra for 1, 2 and 4 DAE molecules coupled with 6EDOT. The coupling of the DAE and 6EDOT generated narrow peaks at ∼1523, ∼1546 and ∼1558 cm^−1^ for the different coupling modes, i.e. 6EDOT-1DAE, 6EDOT-2DAE and 6EDOT-4DAE. The experimentally observed broad Raman peak near ∼1577 cm^−1^ was attributed to stretching vibrations of the two in-plane C=C bonds in DAE and the complicated coupling modes for 6EDOT-1DAE, 6EDOT-2DAE and 6EDOT-4DAE. The strong phonon–phonon coupling affected the configurational entropy of the PEDOT:PSS-*x*DAE thin film and polaron transport. We calculated the ratio of the Raman peak intensities at ∼1577 and ∼1424 cm^−1^, and the result (Fig. S21b) showed that the ratio I_1577_/I_1424_ almost agreed with the results of the thermopower trend for the DAE concentration under UV irradiation. Additionally, the electron paramagnetic resonance spectrum (Fig. 3i) and UV-Vis spectra (Fig. S20b) of the fabricated PEDOT:PSS-38wt%DAE film was not changed by UV-light irradiation, consistent with the almost unchanged electrical conductivity. In other words, modulation of the polaron interfacial occupied entropy with UV light decoupled the strong connection between the thermopower and the electrical conductivity.

### Weak bonding between the DAE and PEDOT

**Figure 4.**
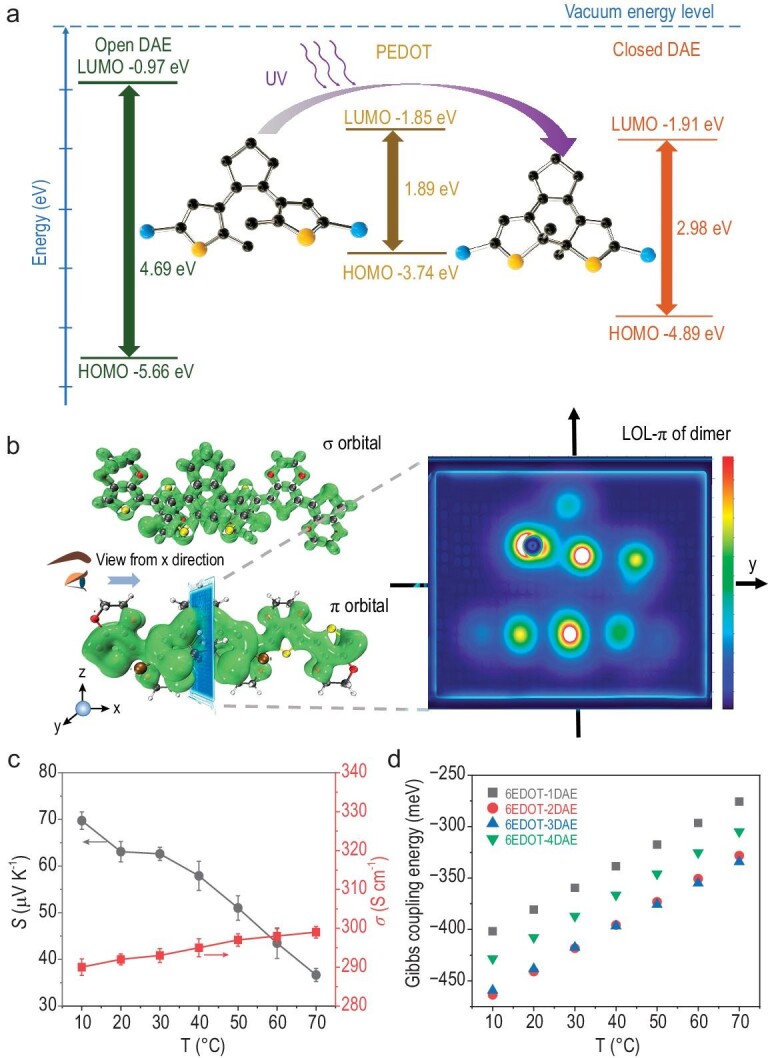
Weak bonding between the DAE and PEDOT. (a) Calculated frontier molecular orbitals (MOs) and energy levels for DAE (in both the open-ring and closed-ring forms) and PEDOT. (b) The DAE and 6EDOT localized σ and π orbital isosurface and cross-sectional view of locator-π (LOL-π). (c) The thermopower and electrical conductivity of PEDOT:PSS-38wt%DAE as a function of temperature. (d) Gibbs coupling energy for 6EDOT-1DAE, 6EDOT-2DAE, 6EDOT-3DAE and 6EDOT-4DAE versus temperature.

To gain better insight into the coupling and polaron interfacial occupied entropy changes between DAE and PEDOT, Density Functional Theory (DFT) calculations were performed to describe the DAE molecular orbitals and energy levels for the open-ring and closed-ring forms. Figure 4a shows the frontier molecular orbitals (FMOs, highest occupied molecular orbital, HOMO, and lowest unoccupied molecular orbital, LUMO) of the DAE and PEDOT. Compared to the open-ring compound (LUMO = −0.97 eV, HOMO = −5.66 eV) formed under visible light, the FMOs of the DAE molecule in the closed-ring form (LUMO = −1.91 eV, HOMO = −4.89 eV) resulting from UV irradiation are closer to the FMOs of PEDOT (LUMO = −1.85 eV, HOMO = −3.74 eV). Most importantly, compared with the open-ring DAE, the HOMO energy level of the closed-ring DAE was obviously higher and closer to the LUMO of PEDOT, and the LUMO of the closed-ring DAE and PEDOT were also much closer in energy, which facilitated polaron flow between the closed-ring DAE and the PEDOT chain.

Figure 4b shows the DAE and 6EDOT localized σ and π orbital isosurfaces and a cross-sectional view of the locator-π (LOL-π). Specifically, π orbital dispersion in molecules indicates the extent of conjugation. The π orbitals of the DAE and 6EDOT dimer were extended over the entire molecular plane, as shown by the isosurface of the localized orbital locator-π (LOL-π), indicating π orbital coupling between the DAE and 6EDOT. In organic molecule systems, the transfer integral *V_e_ or V_h_* (the strength of the electronic coupling between two adjacent molecules; see the supplementary data for computation methodology and details) provides a relatively accurate estimate of *V_ij_* by considering the spatial overlap between the two monomers (monomer *i* and monomer *j*) [51]. Here, we used one, two and four DAE molecules with 6EDOT units to calculate the electron and hole transfer integrals. Fig. S22 shows the optimization charge transfer integral of 6EDOT versus DAE (including the electron transfer integral *V_e_* and the hole transfer integral *V_h_*), and the distance between 6EDOT and DAE was ∼4.2 Å. The calculated electron and hole transfer integrals (Fig. S22) were ∼50 meV, indicating strong electronic coupling between the DAE and the PEDOT chain. As the concentration of DAE was increased, the transfer integrals also increased, consistent with the changes seen in the 1577 cm^−1^ peak in the Raman spectrum shown in Fig. 3g.

Finally, resonant coupling between the DAE and PEDOT was verified by the temperature-dependent thermopower and bonding energy (Fig. 4c and d). Usually, a PEDOT:PSS film shows an increased thermopower and decreased electrical conductivity with increasing temperature (Fig. S16d). However, the UV-induced thermopower and electrical conductivity of the as-fabricated PEDOT:PSS-38wt%DAE showed the opposite trend with a decrease in the thermopower from ∼69.7 to ∼36.6 μV K^−1^ and a slight increase in the electrical conductivity from ∼290.2 to ∼299.2 S cm^−1^ as the temperature was increased from 10 to 70°C (Fig. 4c). The random vibrations disturbed the resonant coupling between DAE and PEDOT. The temperature-dependent Gibbs coupling energy for the DAE molecule and 6EDOT units solidified this explanation (Fig. 4d). The weak bonding between DAE and 6EDOT provided a diversity of interface polaron states or increased the interfacial occupied entropy of the polarons, which increased the thermopower. Our work has elucidated interfacial occupied entropy engineering of the polarons in organic thermoelectric materials. The UV-modulated resonant coupling in the as-fabricated PEDOT:PSS-*x*DAE thin film also provided a platform for directly connecting the temperature gradient, electrical field and light field, which might generate additional new phenomena or applications.

## CONCLUSION

In summary, we have uncovered a direct manipulation method for the polaron interfacial occupied entropy in a PEDOT:PSS thin film through UV-induced resonant coupling between DAE and PEDOT. The UV-light modulation of the polaron interfacial occupied entropy provided a 10-fold thermopower enhancement in the PEDOT:PSS-*x*DAE film, from ∼13.5 to ∼135.4 μV K^−1^, with almost unchanged electrical conductivity, which led to a 93-fold enhancement in the power factor, to ∼527 μW m^−1^ K^−2^. A systematic Raman study and DFT calculations confirmed the resonant coupling between DAE and PEDOT, which generated a broad coupling vibrational peak near 1577 cm^−1^ and a charge transfer integral of −50 meV. Our work provides insight into the decoupling of the connection between the thermopower and the electrical conductivity of an organic thermoelectric film.

## Supplementary Material

nwae009_Supplemental_File
