# Supplementary material for: Polaron interfacial entropy as a route to high thermoelectric performance in DAE-doped PEDOT:PSS films
Source: Natl Sci Rev. 2024 Jan 9;11(3):nwae009. doi: 10.1093/nsr/nwae009 (PMC10858646; doi:10.1093/nsr/nwae009)
Supplement: nwae009_Supplemental_File [file nwae009_supplemental_file.pdf]

Supplementary Materials for

Polaron Interfacial Entropy as a Route to High Thermoelectric Performance in DAE-Doped PEDOT:PSS Films

Jiajia Zhang[†1], Caichao Ye[†1,3], Genwang Wei[1,3], Liang Guo[2], Yuhang Cai[2], Zhi Li[2], Xinzhi Wu[1], Fangyi Sun[1], Qikai Li[1], Yupeng Wang[1], Huan Li[1], Yuchen Li[1], Shuaihua Wang[1], Wei Xu[6], Xuefeng Guo[*,5], Wenqing Zhang*[,1,3], Weishu Liu*[,1,4]

[1]Department of Materials Science and Engineering, Southern University of Science and Technology, Shenzhen, 518055, China
[2]Department of Mechanical and Energy Engineering, Southern University of Science and Technology, Shenzhen, 518055, China
[3]Academy for Advanced Interdisciplinary Studies & Guangdong Provincial Key Laboratory of Computational Science and Material Design, Southern University of Science and Technology, Shenzhen, 518055, China
[4]Guangdong Provincial Key Laboratory of Functional Oxide Materials and Devices, Southern University of Science and Technology, Shenzhen 518055, China
[5]College of Chemistry and Molecular Engineering, National Biomedical Imaging Centre, Peking University, Beijing, China
[6]Beijing National Laboratory for Molecular Sciences, Key Laboratory of Organic Solids, Institute of Chemistry, Chinese Academy of Sciences, Beijing, 100190 China

***Corresponding authors**. Email: liuws@sustech.edu.cn, zhangwq@sustech.edu.cn, guoxf@pku.edu.cn

[†] These authors contributed equally.

1. Materials and Methods

Preparation of PEDOT: PSS-*x*DAE composite films

1,2-Bis(5-chloro-2-methylthiophen-3-yl)cyclopent-1-ene (J&K Scientific, 95%), dimethyl sulfoxide (DMSO, ACS reagent, ≥ 99.9%), and a commercial PEDOT:PSS aqueous solution (PH1000, Clevios) were used directly without further purification. Lithium bis(trifluomethanesulfonyl)imide (Li:TFSI) and L-ascorbic acid were purchased from Aladdin Inc., USA.

First, 1,2-Bis(5-chloro-2-methylthiophen-3-yl)cyclopent-1-ene (DAE) was added into a PEDOT: PSS solution containing 5% DMSO at 80 ℃ with continuous agitation for 1 hour to prepare PEDOT: PSS-*x*DAE (*x* = 16, 28, 38, 44 and 50 wt.%) solutions. Then, glass (typically 1.5 cm × 1.5 cm) substrates were soaked in a mixed solution of hydrogen peroxide and

concentrated sulfuric acid (H_2O_2: H_2SO_4 = 3:7) for 30 min and then washed for 3 min successively with water, ethanol, and acetone in an ultrasonic wave cleaner (60 W). Subsequently, these substrates were dried with an air stream and further cleaned with an O_2 plasma for 10 min. These PEDOT:PSS-*x*DAE solutions were spin-coated onto these glass substrates with an angular speed of 1000 r/min for 60 s to prepare the PEDOT:PSS-*x*DAE thin films. Finally, the PEDOT:PSS-*x*DAE thin films were annealed at 130 ℃ for 10 min. Then, 20% and 40 wt% Li:TFSI and L-ascorbic acid were added into the PEDOT:PSS-38wt.% DAE solution to adjust the doping level, and the films were prepared as described above.

Performance of the PEDOT:PSS-*x*DAE thin films

The thermopower (S) was measured with a self-built system containing two Peltier devices. The thermovoltages (ΔV) and temperature gradients (ΔT) were detected with a Keithley 2000 multimeter and two T-type thermocouples (Omega, US). The electrical conductivities (σ) were measured with the 4-point probe technique with a Keithley 2400 source/meter. The gold electrodes were sputtered deposition by DM200 sputtering instrument. The electrical contacts were made with thin copper sheets connected with silver conductive paint (SCP03B, Electrolube). The 355 nm UV light source was a Talon-355-15NL laser. The 365 nm and 395 nm UV light source were purchased from PURI Materials Co. Ltd. A Countor GT K 3D optical microscope was used to measure the thicknesses of the PEDOT:PSS-*x*DAE thin films. X-ray diffraction (XRD) patterns were measured with a D/max2500 (Rigaku, Japan) diffractometer operated with a 40 kV voltage and a 200 mA current and a Cu–Kα radiation source (κ = 1.54 Å). The Raman spectrum was measured with a LabRAM HR Evolution microscopic confocal Raman spectrometer (HORIBA Scientific); the visible laser generated radiation with λ = 532 nm, and the UV laser generated radiation with λ = 325 nm. The EPR measurement was performed with a Bruker Elexsys E500 spectrometer operating at 9.88 GHz (X-band).

DFT simulations

Theoretical methodology

The transfer integral V represents the strength of electronic coupling between adjacent molecules. It is calculated with the direct coupling approach, which provides a relatively accurate estimate for V_{ij} by considering the spatial overlap between two monomers (monomer i and monomer j) [1]. In this scheme, the electron coupling is given by

$$V_{ij} = \frac{h_{ij} - \frac{1}{2}(h_{ii} + h_{jj})S_{ij}}{1 - {S_{ij}}^2}$$

where $h_{ij} = \langle \varphi_i | h_{KS} | \varphi_j \rangle$, $S_{ij} = \langle \varphi_i | S | \varphi_j \rangle$, and $|\varphi_i\rangle$ is the wave function for the frontier molecular orbital of the *i or j* monomer. h_{ij} is the charge transfer integral, and S_{ij} is the spatial overlap integral. h_{KS} is the Kohn–Sham Hamiltonian of the dimer system, which was calculated with the following equation: $h_{KS} = SC\varepsilon C^{-1}$, where S is the intermolecular overlap matrix and C and ε are the molecular orbital coefficients and energies from one-step diagonalization without iteration.

Computational details

The calculations of the transfer integral, optimized structures, and molecular orbital energy levels for DAE and six EDOT unit systems were performed with the B3LYP [2]correlation functionals with the 6-311G(d,p) [3-5] basis set and the site-energy corrected method, which has been proven to give good descriptions of the transfer integrals at the DFT level. The Raman spectra were simulated with a combination of empirical potentials and first-principles calculations using B3LYP at the 6-31G(d) level [3,5,6] with a Raman frequency correction factor of 0.9614 [7], which made it easy to investigate the weak interactions between molecules. These DFT-D3(BJ) [8,9] corrections provided satisfactory results that fit well with the experimental findings. The structural optimizations and Raman simulations of the molecules were performed with the Gaussian 16 program [10]. The molecular orbital calculations were performed with the Multiwfn package [11]. The Gibbs coupling energy of DAE and the PEDOT system was calculated based on the definition $G_{coupling\ (DAE\&PEDOT)} = G_{(DAE\&PEDOT)} - G_{(DAE)} - G_{(PEDOT)}$, where $G_{(DAE\&PEDOT)}$ is the DFT total Gibbs free energy of the corresponding system for coupled DAE&PEDOT, and $G_{(DAE)}$ and $G_{(PEDOT)}$ represent the DFT Gibbs free energy of the isolated DAE and the corresponding PEDOT molecule, respectively. According to this definition, a large negative value for $G_{coupling}$ means stable and strong coupling.

2. Degeneracy definition of the PEDOT:PSS-*x*DAE film

The closed-ring form of diarylethene and PEDOT share similar C-C=C-C carbon skeletons and easily couple with each other to generate a new polaron interface state, which increases the thermopower. Every DAE molecule coupled with a PEDOT chain forms a new polaron interface state. When DAE molecules couple with the PEDOT chains, they release energy (Gibbs coupling

energy) to achieve thermodynamic equilibrium. The lower the Gibbs coupling energy is, the more stable the system. We calculated the Gibbs coupling energy and the HOMO, and LUMO of the DAE and 6EDOT chains at different configurations (Fig. S1-4, Table S1-4). We set the differences in the Gibbs coupling energy for DAE molecules and PEDOT in their most stable configurations to be less than 1 k_BT (approximately 23.55 meV near room temperature) and that the polaron interfacial state at the same energy level (HOMO level for a p-type material here, less than 1 k_BT, approximately 23.55 meV near room temperature) as degenerate. According to Fig. S1 and Table S1, although the Gibbs coupling energies of 1DAE-2 and 1DAE-3 were close, the HOMO level difference was over 1 k_BT, so the degeneracy number was 1 for one DAE molecule versus EDOT units. When two DAE molecules were coupled with six EDOT units (as shown in Fig. S2 and Table S2), 2DAE-1, 2DAE-2, and 2DAE-3 were almost the same states, so the degeneracy number was 3 for two DAE molecules coupled with six EDOT units. Because 3DAE-1, 3DAE-2, and 3DAE-3 are almost the same states (Fig. S3 and Table S3), the degeneracy was also 3 for three DAE molecules versus six EDOT units. For the system with four DAE molecules versus six EDOT units (shown in Fig. S4 and Table S4), only 4DAE-1 and 4DAE-6 are in almost the same states, so the degeneracy number is 2.

3. Relationship between thermopower and the polaron interface occupied entropy

According to statistical thermodynamics, entropy is a logarithmic measure of the number of system states with significant occupation probabilities [12]:

$$\hat{S} = -k_B \sum_i p_i ln p_i \qquad (1)$$

p_i is the probability that the system is in the ith state, and k_B is the Boltzmann constant.

For organic thermoelectric materials, the flow of charge carriers (polarons) along the temperature gradient $-\nabla T$ leads to an entropy change that determines the thermopower ($S = \hat{S} / e$). The polaron transport entropy is associated with the disorder or randomness of the hopping processes. It represents the degree of uncertainty or multiplicity of available states available to the charge carriers during transport. Coupled small molecule doping of the conducting polymers could form new polaron interfacial states between the polymer chains and small molecules, which would provide extra polaron sites at the interface and form a new interfacial polaron state,

directly increasing the interfacial entropy of the polaron. In PEDOT:PSS-*x*DAE, the addition of DAE results in polaron interface states between DAE and the PEDOT chains, which causes the polarons to occupy new sites. The thermopower of PEDOT:PSS-*x*DAE was much larger than that of PEDOT:PSS because the high interfacial state degeneracy increased the interfacial-polaron entropy, while the presence of extra polaron-occupied sites modified the energy levels and increased the density of states (Fig. S5). A higher density of available energy levels enables more efficient energy conversion and transport processes, leading to a larger thermopower.

There are two types of polarons in PEDOT:PSS-*x*DAE: interfacial localized polarons and noninterfacial-localized polarons. The probabilities of interfacial localized polarons and noninterfacial localized polarons in PEDOT:PSS-*x*DAE are denoted by *f* and *1-f*, respectively.

The polaron entropy can be expressed as follows:

$$\hat{S}_{P-interf} = -k_B n f \ln f - k_B(1-f)\ln(1-f) \qquad (2)$$

where *f* represents the probabilities of interfacial localized polarons, and *n* is the degeneracy of the localized polarons at the interface states (*n* definitions are shown in the SI-2 section).

Due to calculation limitations and ideal calculation models, we selected six EDOT units to study the coupled states with different molar ratios of DAE molecules. For example, one DAE molecule can only form an interface state with six EDOT units (Fig. S1), so the coverage of DAE versus 6EDOT is $y=m/6$, and *m* represents the number of DAE molecules. For the interfacial localized polaron probabilities $f=y$, the increasing thermopower ($S_{P-interf}$) caused by the polaron interfacial occupied entropy can be expressed as follows:

$$S_{P-interf} = \frac{\hat{S}_{P-interf}}{e} = -\frac{k_B}{e}(ny\ln y + (1-y)\ln(1-y)) \qquad (3)$$

4. Density of states calculation for the PEDOT:PSS-*x*DAE film

With the DOS calculations (Fig. S5) of the DAE molecules and 6EDOT-*x*DAE, we observed that the DOS for 6EDOT-*x*DAE increased as the DAE concentration increased to near the Fermi level (HOMO). This indicated the formation of new polaron interfacial states, which increased the DOS near the Fermi level, allowed for more efficient energy conversion and transport processes, and increased the thermopower of the PEDOT-*x*DAE film.

5. Thermoelectric measurements of the PEDOT:PSS-xDAE and PEDOT:PSS materials

The thermopower measurements were conducted according to the definition, i.e., $S= -(V_H-V_C)/(T_H-T_C)$, where $V_H – V_C$ is the voltage difference and $T_H – T_C$ is the temperature difference. Fig. S6 (a-c) shows a thermopower schematic illustration for the as-prepared PEDOT:PSS-*x*DAE film under UV illumination.

We also prepared a series of different thickness PEDOT-38wt.%DAE film, then we measured the thermopower before and after the UV light condition. Below the 200 nm, the enhanced thermopower almost keep stable at about 132 μV K^{-1}. As the thickness further increased from 200 nm to 10 μm, the thermopower decreased from 132 to 15 μV K^{-1} (shown in Fig. S12). We think the UV light has limited penetration depth, so it hardly occurs the closed-ring reaction in the deeper place for the bulk PEODT:PSS-*x*DAE materials. Therefore, the UV light illumination strategy can't work for bulk organic materials.

6. Structural characterization of the PEDOT:PSS-*x*DAE films

The XRD patterns measured for the PEDOT:PSS-38wt.%DAE films before and after UV irradiation (shown in Fig. S17) did not differ, which means that the arrangement of the PEDOT chains was relatively stable. In the UV–visible-NIR absorption spectra, $\pi \rightarrow \pi^*$ electron hopping generated a strong absorption band (namely, K-band) in the 210–250 nm range for a conjugated compound. With increasing conjugation, the K-band absorption peak was redshifted. In Fig. S18, the K-band absorption peak for the PEDOT:PSS-*x*DAE (*x*=38 wt.%) film redshifted from 210 nm to 229 nm upon UV light irradiation. This illustrated that UV light irradiation increased the conjugation of the PEDOT:PSS-*x*DAE films, which induced coupling of the DAE and the PEDOT chains and produced new polaron interface states to enhance the thermoelectric performance.

7. Electron and hole transfer integrals of the PEDOT:PSS-*x*DAE film

In an organic molecule system, the transfer integral V_e *or* V_h (the strength of electronic coupling between two adjacent molecules) provides a relatively accurate estimate of V_{ij} due to spatial overlap between the two monomers (monomer *i* and monomer *j*). Here, we used one, two, and four DAE molecules and six EDOT units to calculate the electron and hole transfer integrals. The calculated electron transfer integrals (Fig. S21) were 49.53 meV, 55.66 meV, and 44.42 meV for one, two, and four DAE molecules versus six EDOT units, respectively. The calculated hole transfer integrals (Fig. S20) were 40.70 meV, 46.93 meV, and 38.04 meV for one, two, and four DAE molecules versus six EDOT units, respectively. The charge transfer integrals indicated strong electronic coupling between the DAE and the PEDOT chains, which resulted in new polaron states for the interface between the DAE molecules and the PEDOT chain. As the concentration of DAE was increased, the transfer integrals first increased and then decreased, which was consistent with the dependence of the thermopower on the DAE concentration, as shown in Fig. 2a.

References

1 Valeev E, Coropceanu V, Filho, D *et al.* Effect of electronic polarization on charge-transport parameters in molecular organic semiconductors. *J Am Chem Soc* 2006; **128**: 9882-6.

2 Stephens P, Devlin F, Chabalowski C *et al.* Ab Initio Calculation of Vibrational Absorption and Circular Dichroism Spectra Using Density Functional Force Fields. *J Phys Chem* 1994; **98**: 11623-7.

3 Hariharan C, Pople J. Accuracy of AHn equilibrium geometries by single determinant molecular orbital theory. *Mol Phys* 1974; **27**: 209-14.

4 Krishnan R, Binkley J, Seeger R *et al.* Self-consistent molecular orbital methods. XX. A basis set for correlated wave functions. *J Chem Phys* 1980; **72**: 650-4.

5 Petersson G, Bennett A, Tensfeldt T *et al.* A complete basis set model chemistry. I. The total energies of closed□shell atoms and hydrides of the first□row elements. *J Chem Phys* 1988; **89**:2193-218.

6 Rassolov V, Pople J, Ratner M *et al.* 6-31G[*] basis set for atoms K through Zn. *J Chem Phys* 1998; **109**: 1223-9.

7 Scott A, Radom L. Harmonic vibrational frequencies: An evaluation of hartree−fock, møller−plesset, quadratic configuration interaction, density functional theory, and semiempirical scale factors. *J Phys Chem* 1996; **100**: 16502-13.

8 Grimme S, Antony J, Ehrlich S *et al.* A consistent and accurate ab initio parametrization of density functional dispersion correction (DFT-D) for the 94 elements H-Pu. *J Chem Phy* 2010; **132**: 154104.

9 Grimme S, Ehrlich S, Goerigk L. Effect of the damping function in dispersion corrected density functional theory. *J Comput Chem* 2011; **32**: 1456-65.

10 Gaussian 16 Rev. C.01 (Wallingford, CT, 2016).

11 Lu T, Chen F. Multiwfn: A multifunctional wavefunction analyzer. *J Comput Chem* 2012; **33**: 580-92.

12 Zhou F, Maxisch T, Ceder G. Configurational electronic entropy and the phase diagram of mixed-valence oxides: the case of Li_xFePO_4. *Phys Rev Lett* 2006; **97**: 155704.

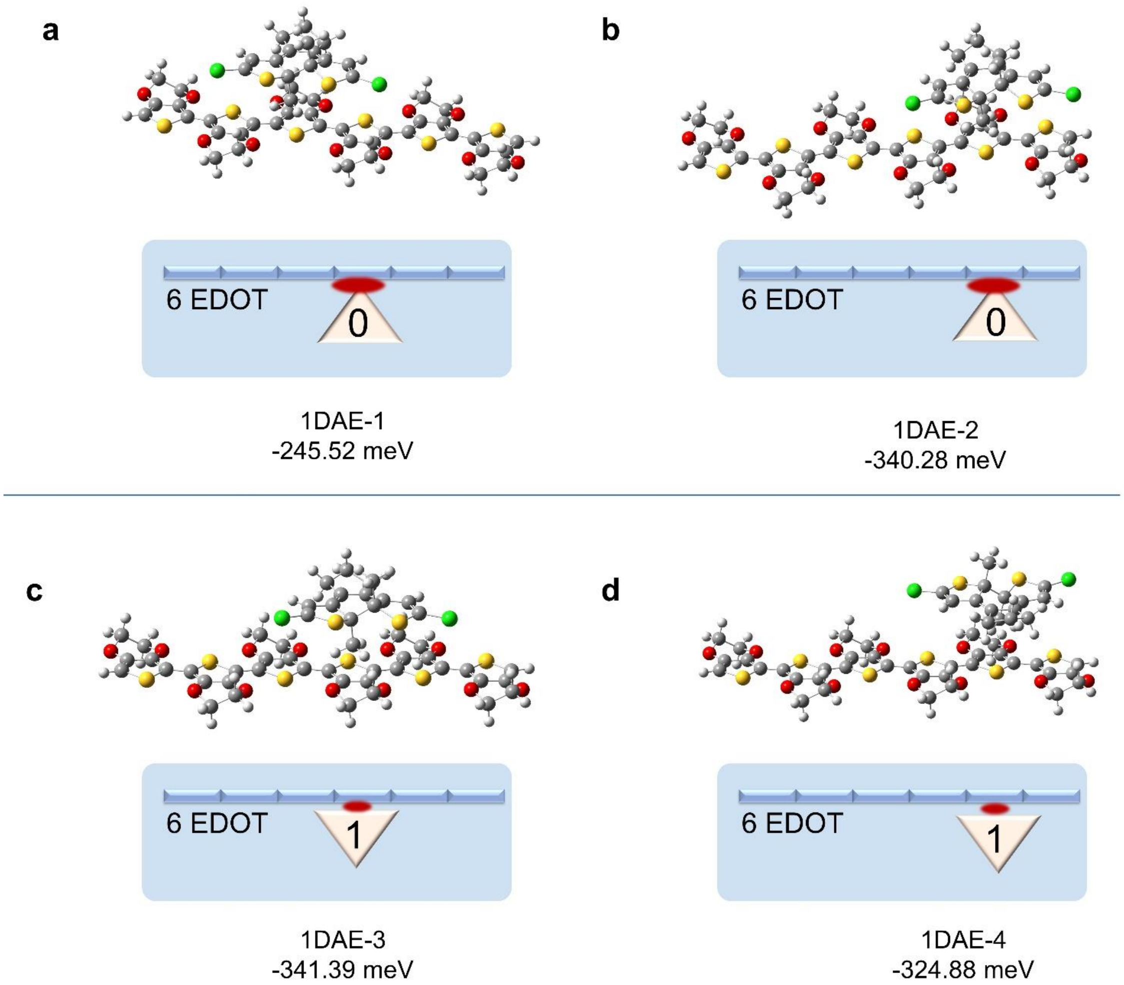

Fig. S1. (a-d) Coupling configurational diagrams for one DAE molecule versus six EDOT units.

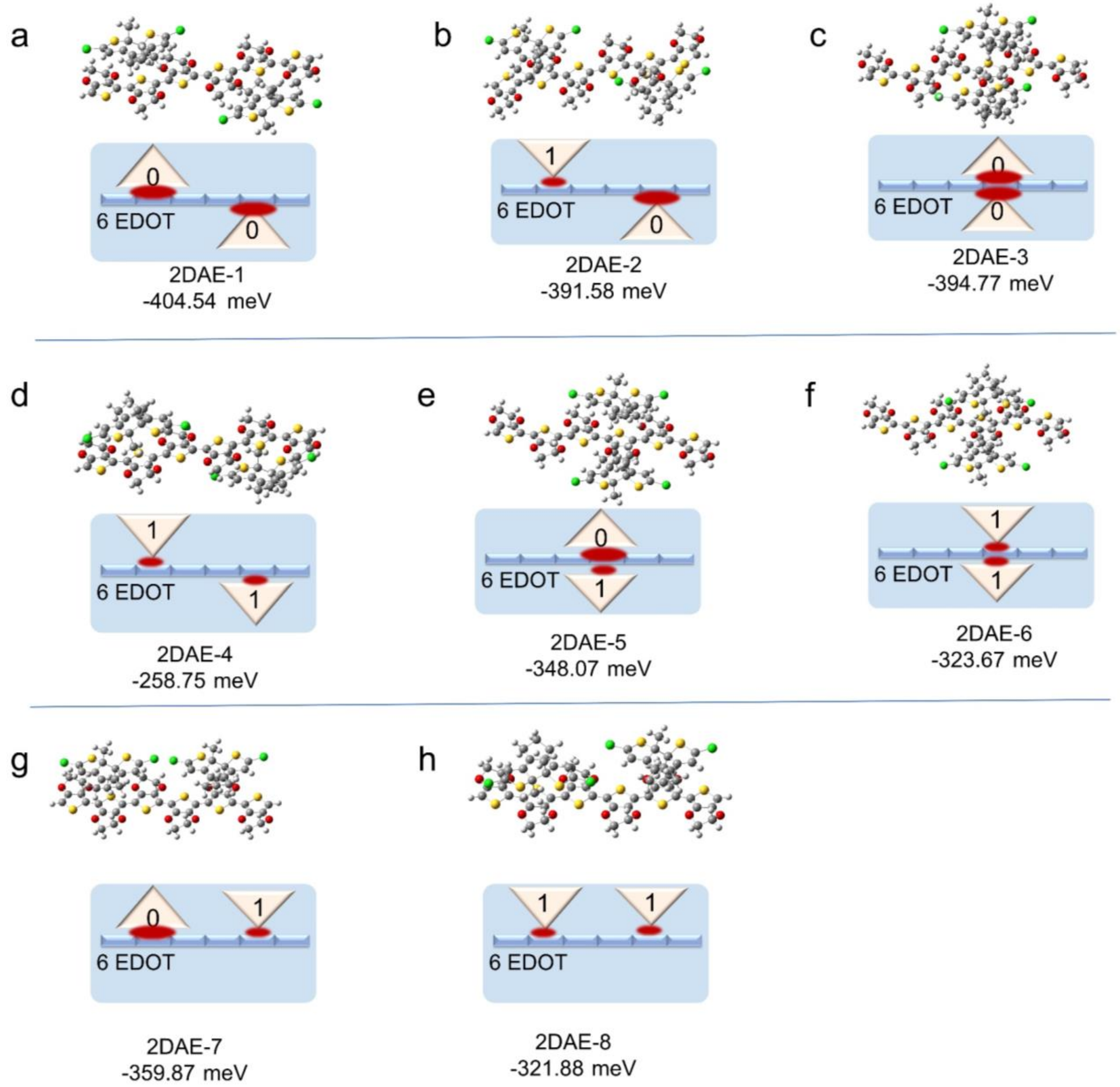

Fig. S2. (a-i) Coupling configurational diagrams for two DAE molecules versus six EDOT units.

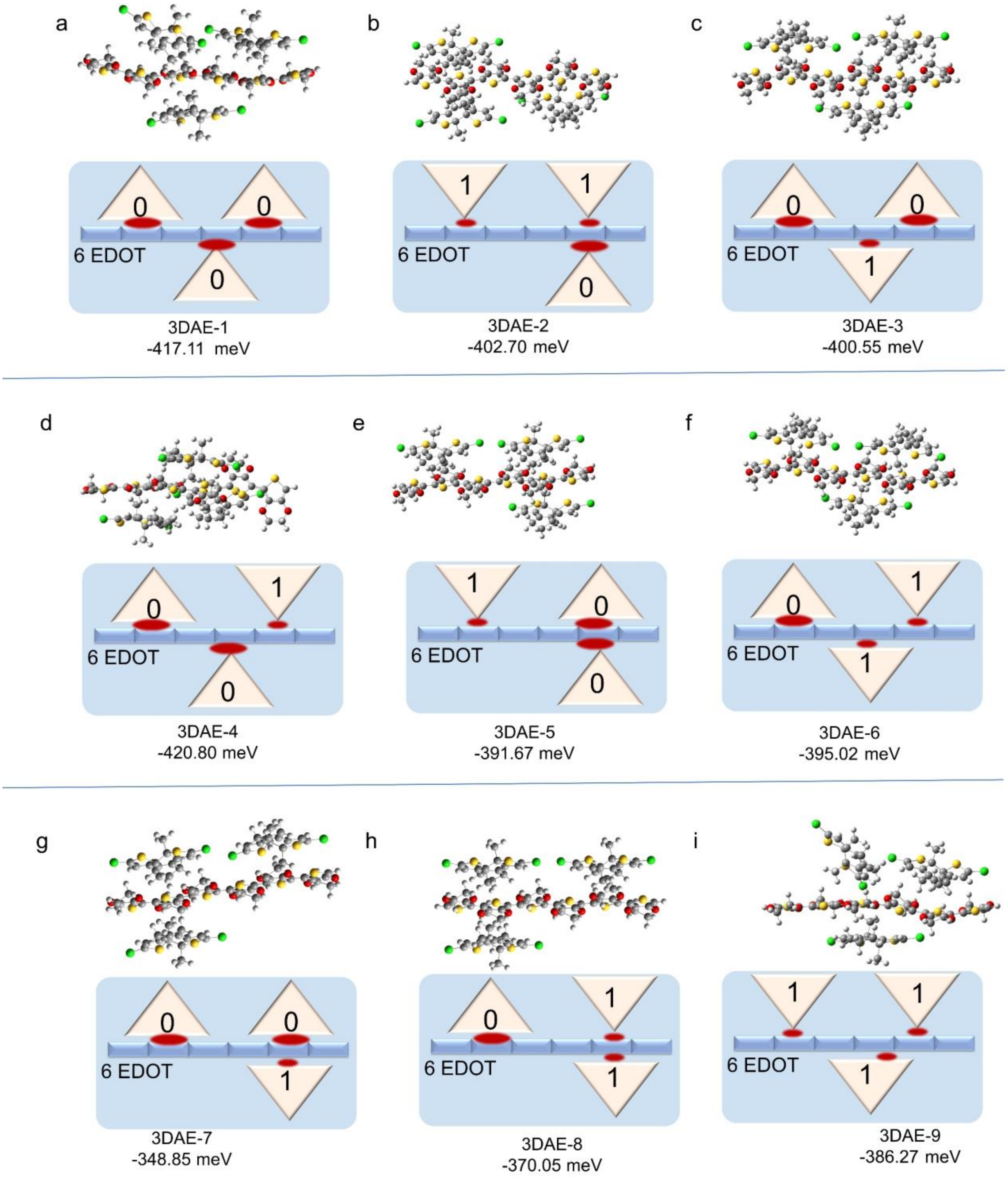

Fig. S3. (a-i) Coupling configurational diagrams for three DAE molecules versus six EDOT units.

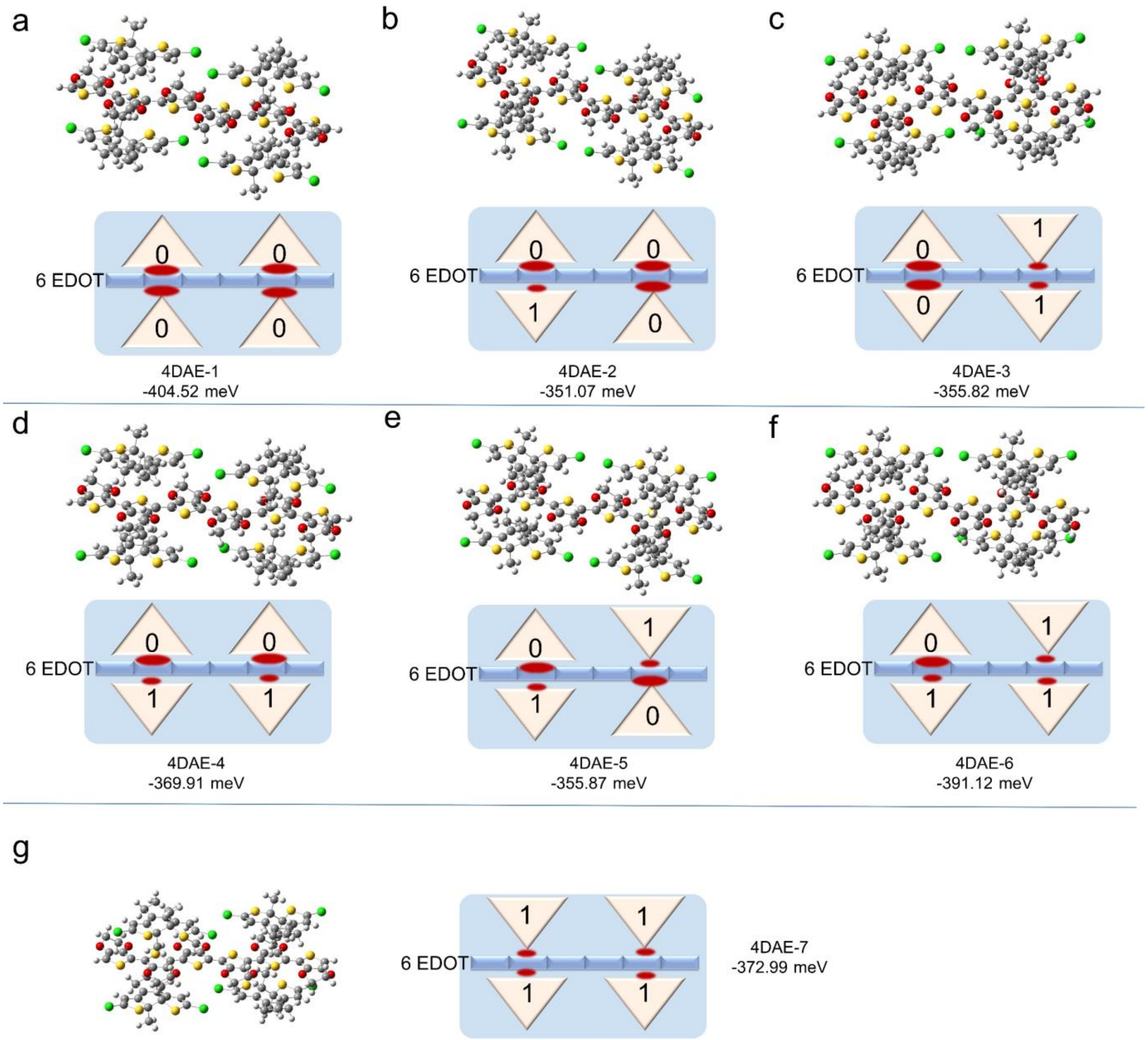

Fig. S4. (a-g) Coupling configurational diagrams for four DAE molecules versus six EDOT units.

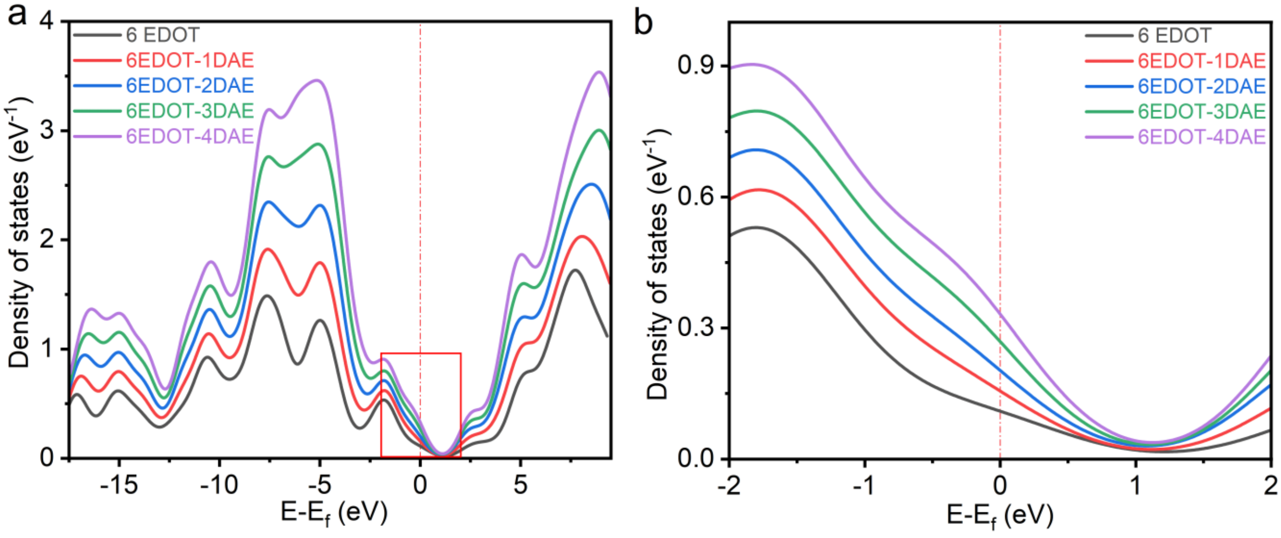

Fig. S5. (a-b) Density of states for 6EDOT and coupling of one, two, three, and four DAE molecules with six EDOT units.

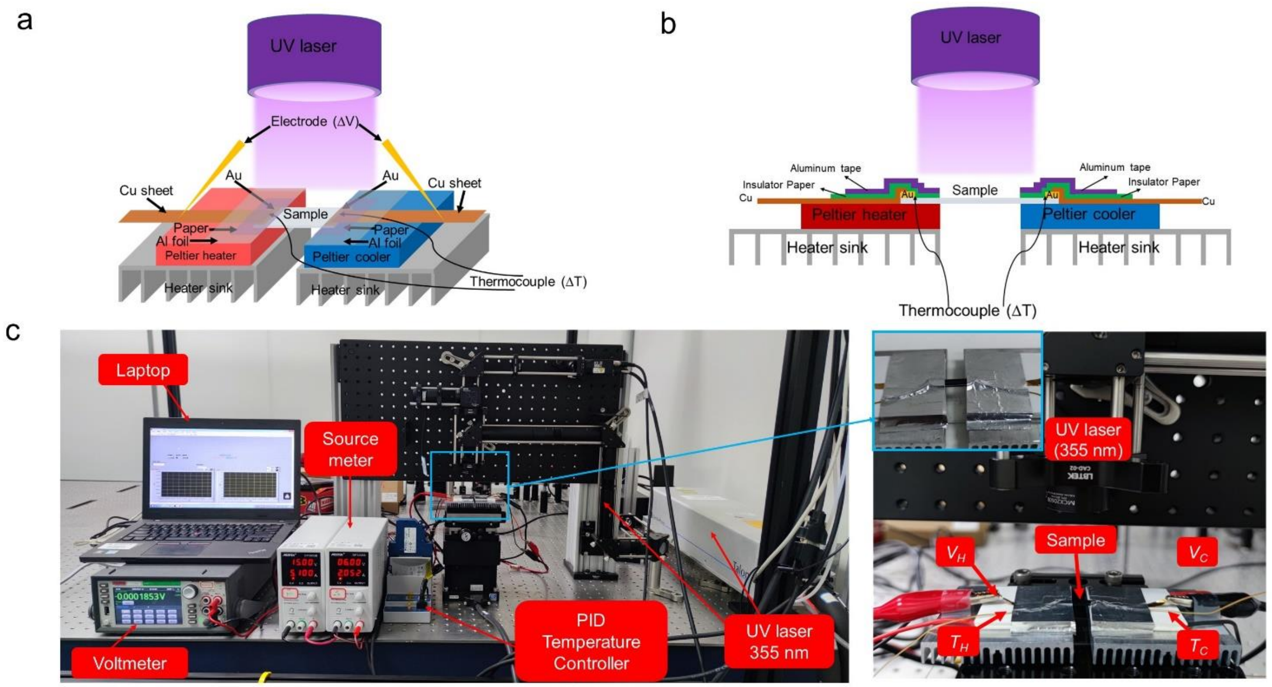

Fig. S6. (**a**) Thermopower schematic illustration of the as-prepared PEDOT:PSS-*x*DAE film under UV illumination. (**b**) *Cross-sectional view (not drawn to scale and proportion)* **(c)** Thermopower measured equipment of the as-prepared PEDOT:PSS-*x*DAE film under UV

illumination.

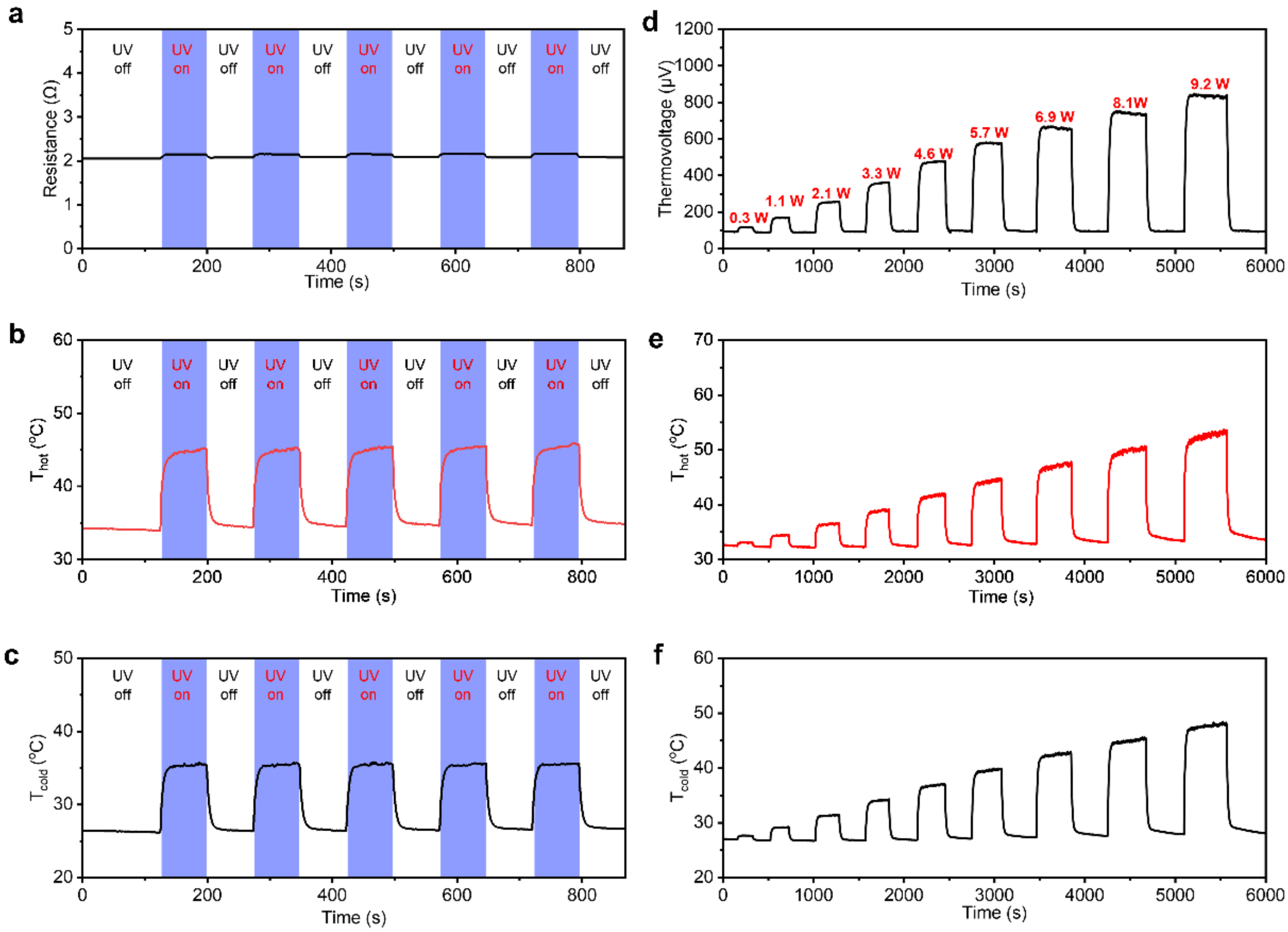

Fig. S7. (a-c) Resistance and temperature for hot and cold end evolution of a PEDOT:PSS-*x*DAE (*x*=38 wt.%) sample versus time, which were generated by turning the UV light on or off (the UV laser power was 1.3 W cm^{-2}). **(d-f)** Thermovoltage and temperature for hot- and cold-end evolution of a PEDOT:PSS-*x*DAE (*x*=38 wt.%) sample, which was generated by turning the UV light on or off with different UV laser powers.

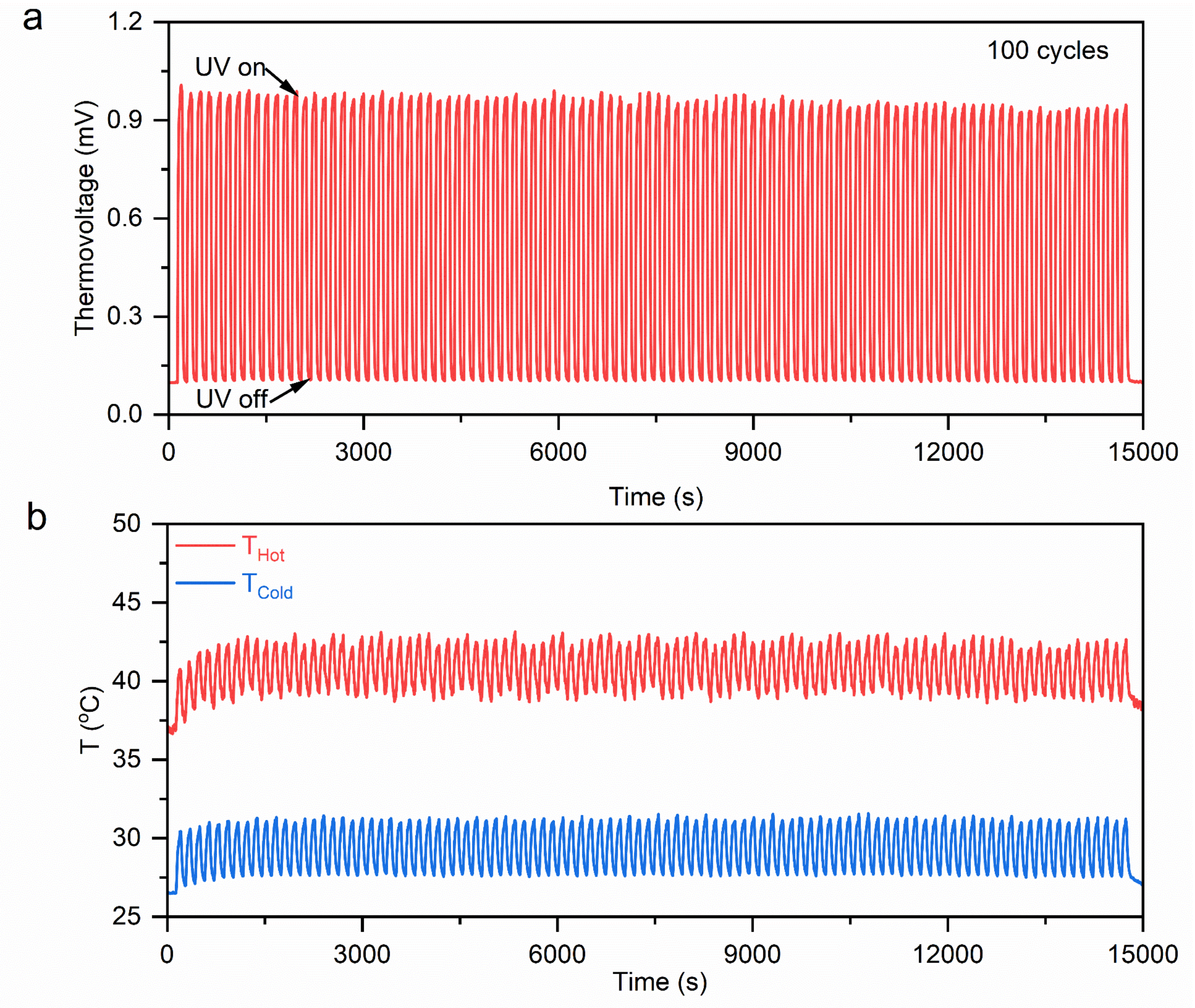

Fig. S8. (a-b) Thermovoltage and temperature for hot and cold end evolution of a PEDOT:PSS-*x*DAE (*x*=38 wt.%) sample versus time, which were generated by turning the UV light on or off (the UV laser power was 1.3 W cm^{-2}).

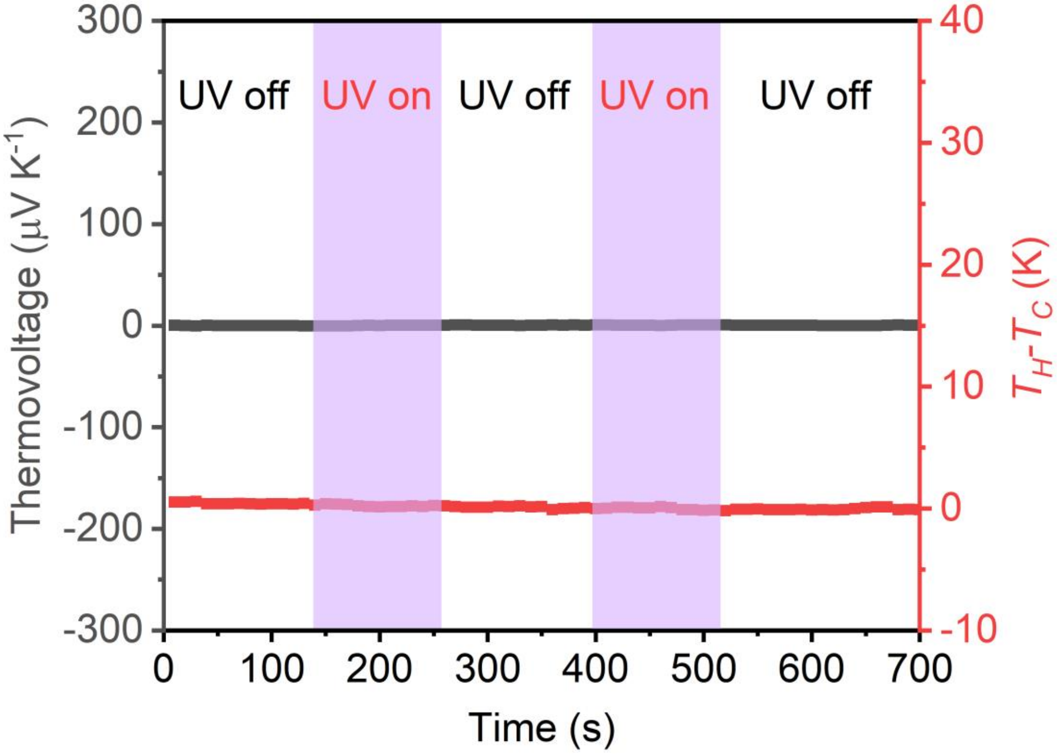

Fig.S9 Thermovoltage and temperature gradient for hot and cold end evolution of a PEDOT:PSS-*x*DAE (*x*=38 wt.%) sample versus time, which were generated by turning the UV light on or off (the UV laser power was 0.8 W cm^{-2}, the wavelength is 365 nm).

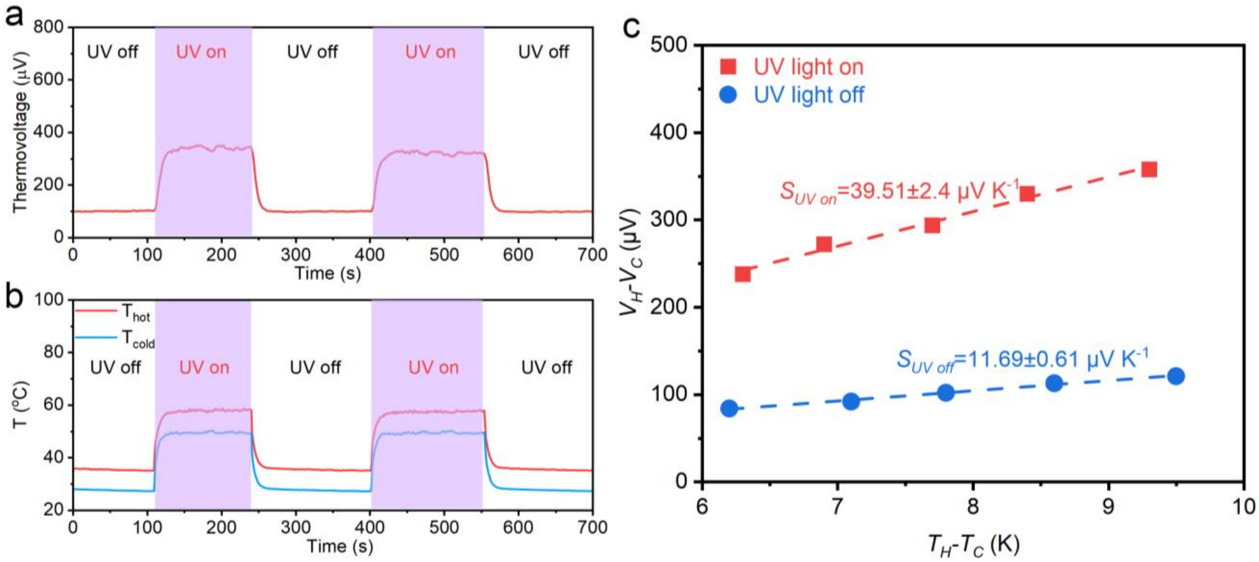

Fig. S10 Thermovoltage and temperature for hot and cold end evolution of a PEDOT:PSS-*x*DAE (*x*=38 wt.%) sample versus time, which were generated by turning the UV light on or off (the UV laser power was 0.8 W cm^{-2}, the wavelength is 365 nm).

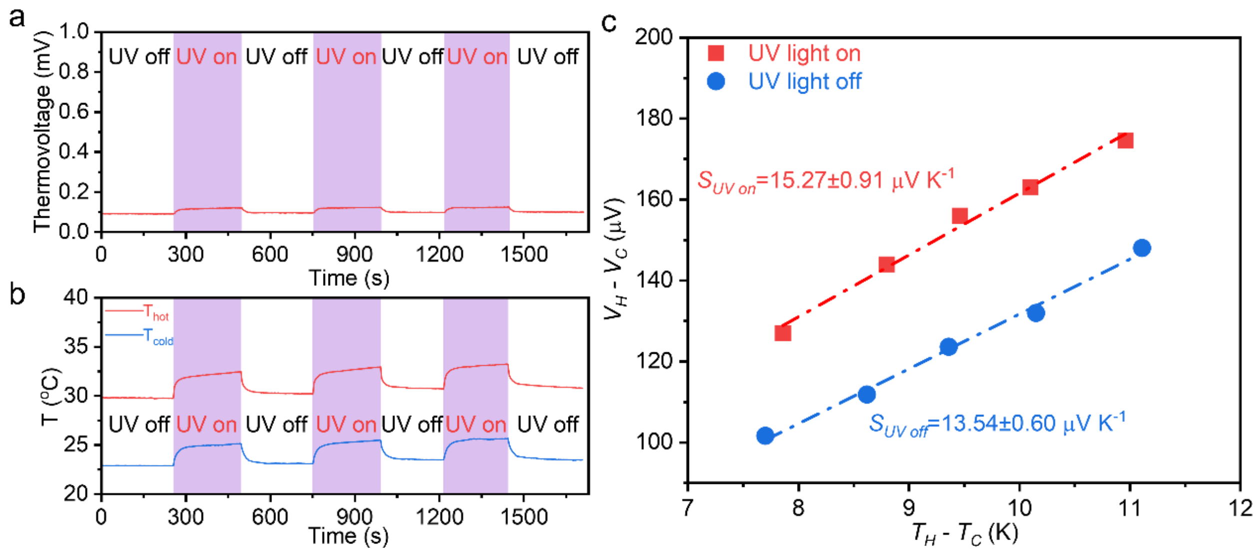

Fig. S11 Thermovoltage and temperature for hot and cold end evolution of a PEDOT:PSS-*x*DAE (*x*=38 wt.%) sample versus time, which were generated by turning the UV light on or off (the UV laser power was 0.8 W cm^{-2}, the wavelength is 395 nm).

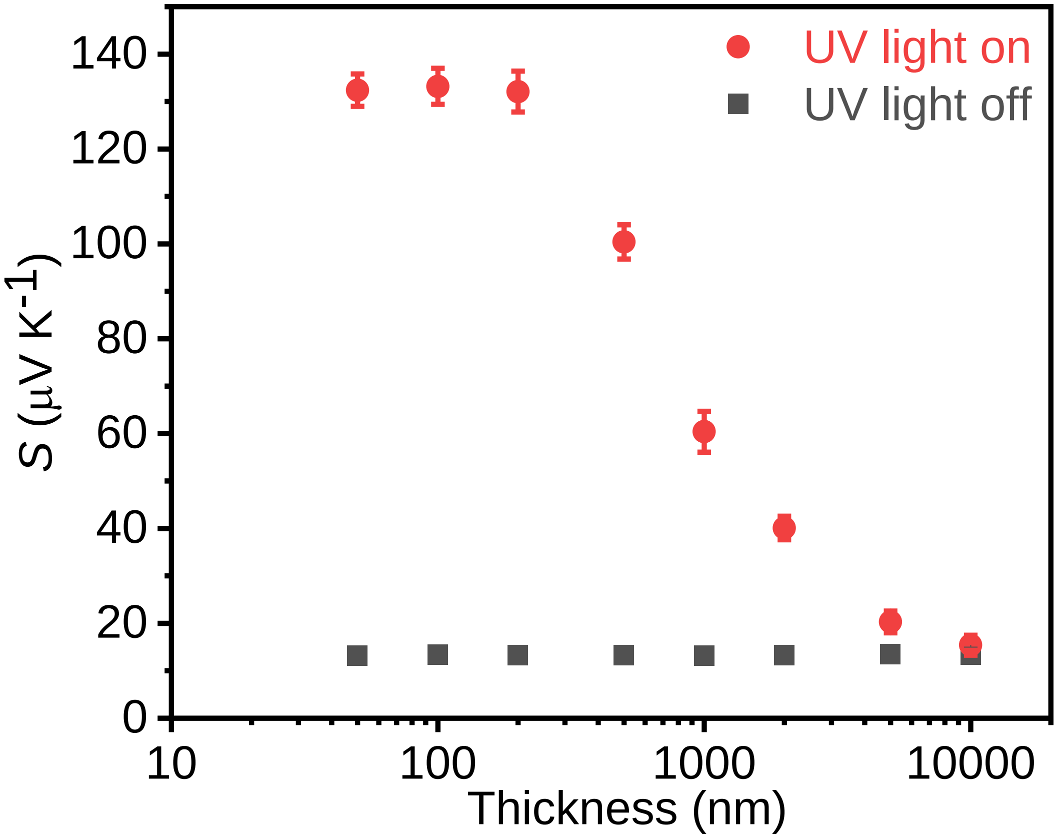

Fig. S12 The thermopower versus thickness relationship for the PEDOT:PSS-38wt.% DAE film.

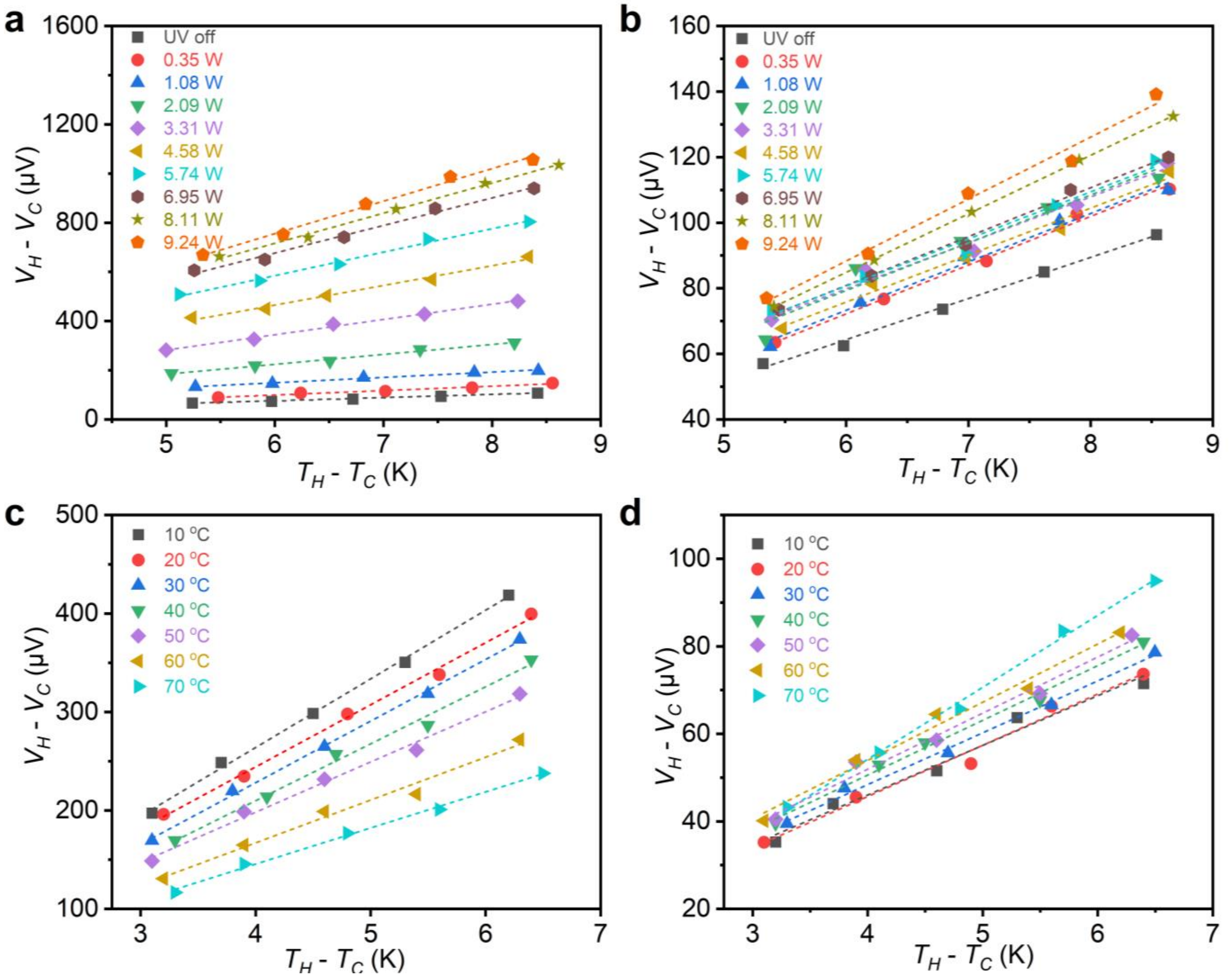

Fig. S13. Thermopower measurements for PEDOT:PSS-*x*DAE (*x*=38 wt.%) (**a** and **c**) and PEDOT:PSS (**b** and **d**) materials with different UV laser powers and temperatures. $V_H - V_C$ is the voltage difference, while $T_H - T_C$ is the temperature difference.

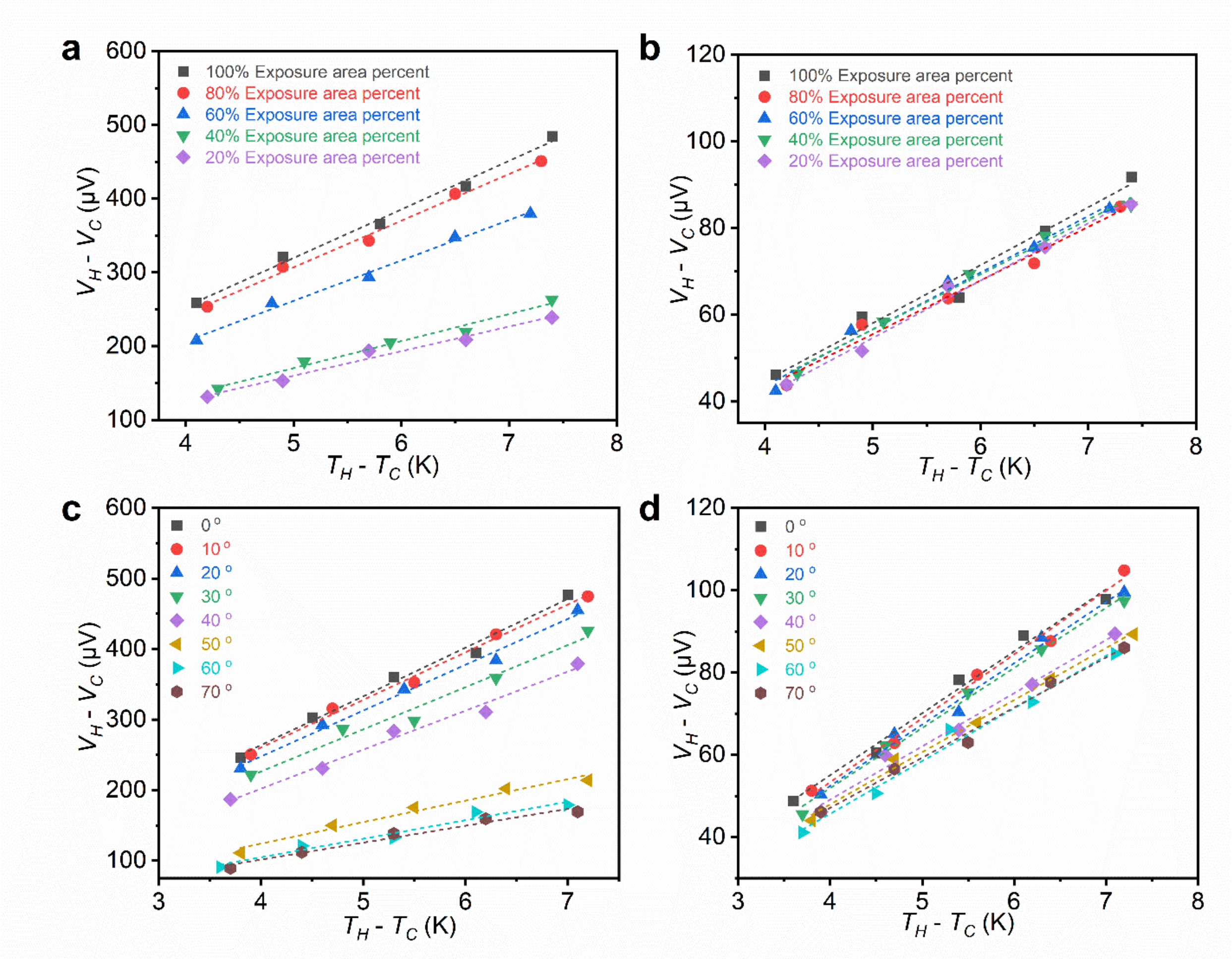

Fig. S14. Thermopower measurements for the PEDOT:PSS-*x*DAE (*x*=38 wt.%) (**a** and **c**) and PEDOT:PSS (**b** and **d**) materials with different angles of light incidence (UV power=0.6 W cm^{-2}) and exposure area percentages (the UV power=0.6 W cm^{-2}). $V_H - V_C$ is the voltage difference, while $T_H - T_C$ is the temperature difference.

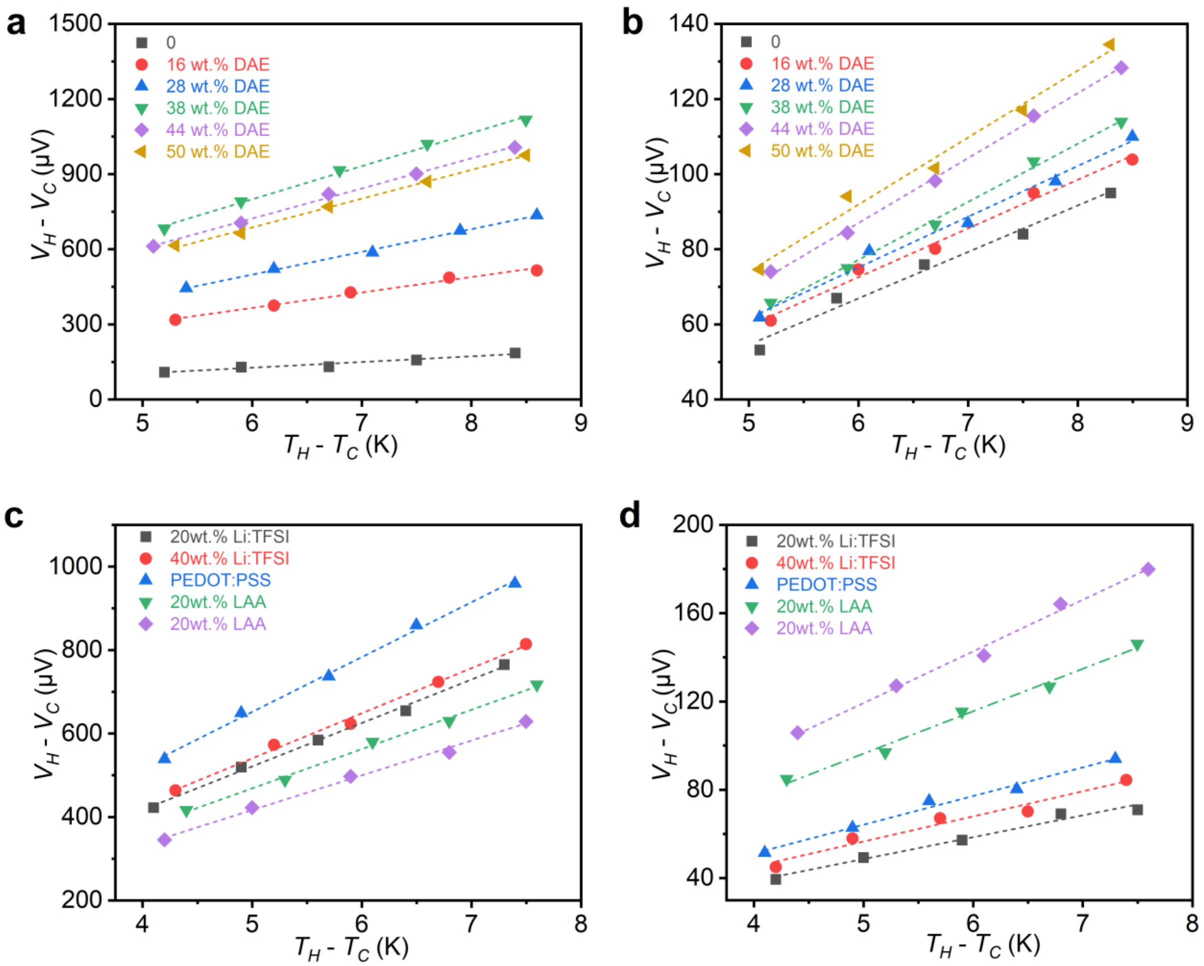

Fig. S15. Thermopower measurements for the PEDOT:PSS-*x*DAE (*x*=38 wt.%) (**a** and **b**) and PEDOT:PSS-*x*DAE (*x*=38 wt.%) (**c** and **d**) materials with different DAE concentrations and doping levels with the ionic liquid Li：TFSI and dedoping with LAA (the UV power=1.3 W cm^{-2}). $V_H - V_C$ is the voltage difference, while $T_H - T_C$ is the temperature difference.

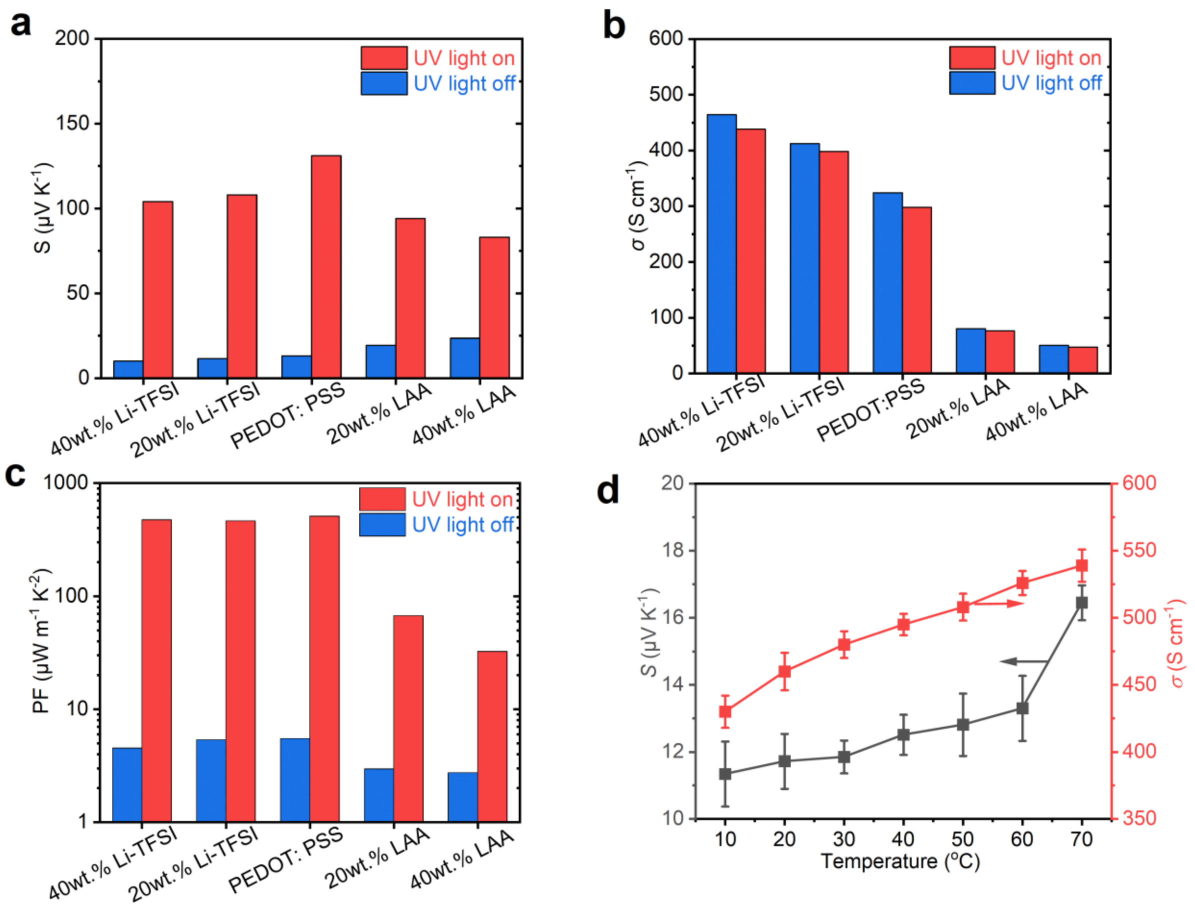

Fig. S16. (a-c) Thermopower, conductivity, and power factor of PEDOT:PSS-*x*DAE (*x*-38 wt.%) films with the UV light on (UV power density at 1.3 W cm^{-2}) or off after doping with the ionic liquid Li：TFSI and dedoping with LAA. (**d**) Dependence of the thermopower and electrical conductivity of PEDOT:PSS on temperature.

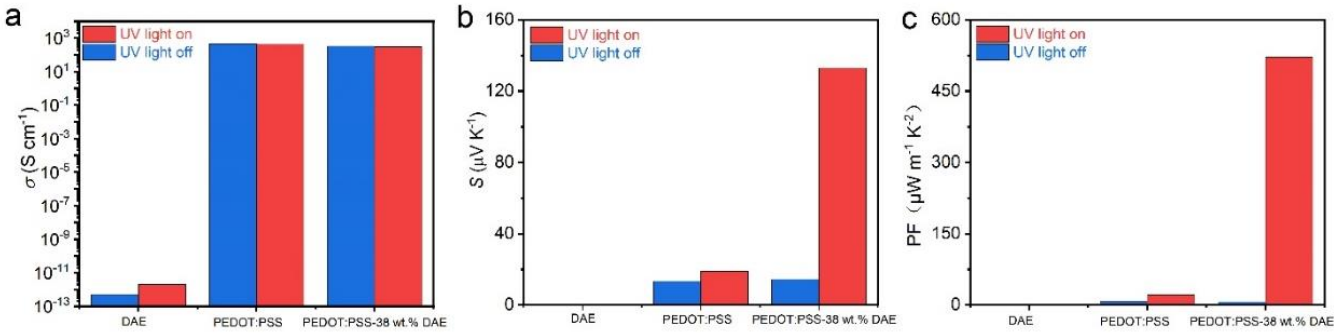

Fig. S17 (a-c) Comparison of the thermopower, electrical conductivity, and power factor of pure DAE film (~ 500 μm), PEDOT:PSS, and PEDOT:PSS-38wt.% DAE films with UV light off and with UV light on (UV power = 1.3 W cm^{-2}).

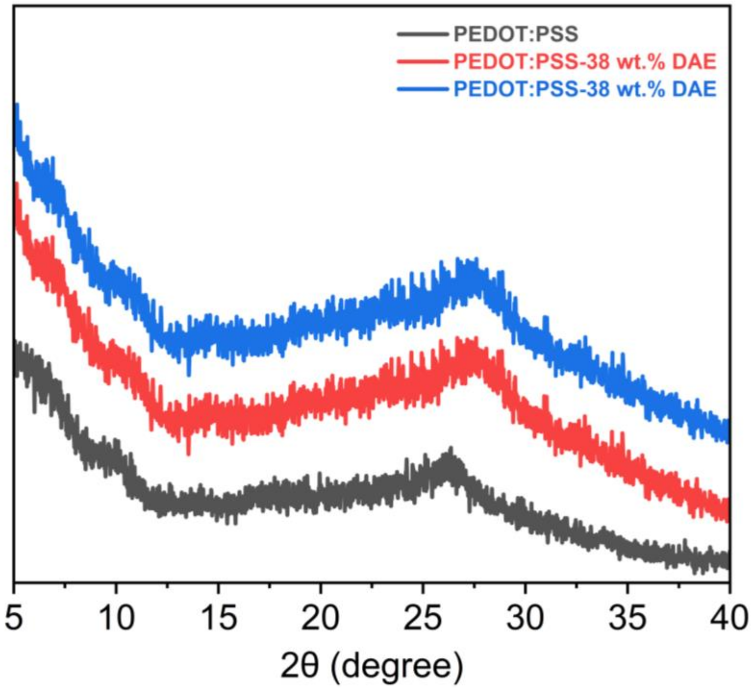

Fig. S18. X-ray diffraction data for the PEDOT:PSS and PEDOT:PSS-38 wt.%DAE films with the UV light off and on.

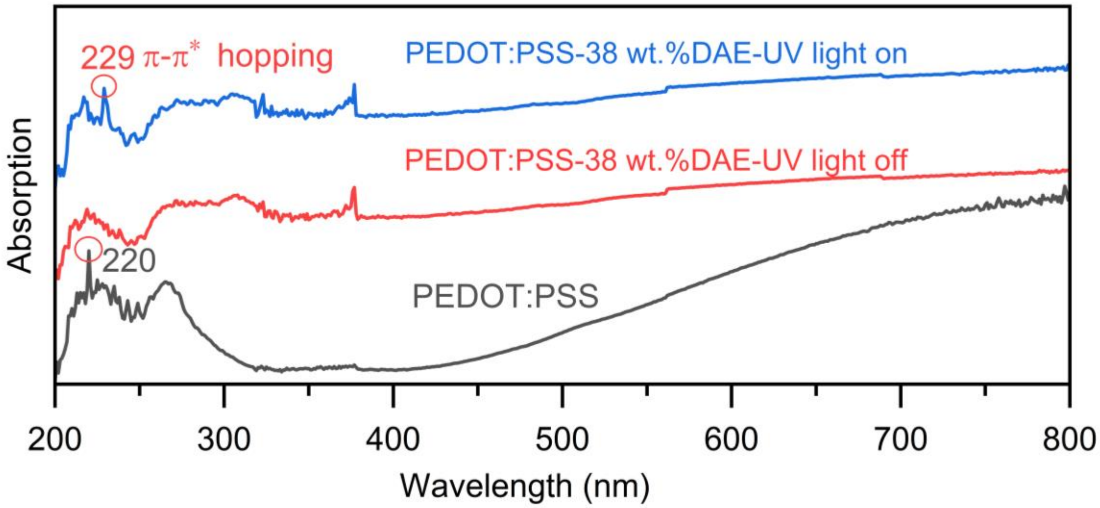

Fig. S19. UV-visible-NIR absorption spectra of the PEDOT:PSS and PEDOT:PSS-*x*DAE (*x*=38 wt.%) films measured before and immediately after UV irradiation.

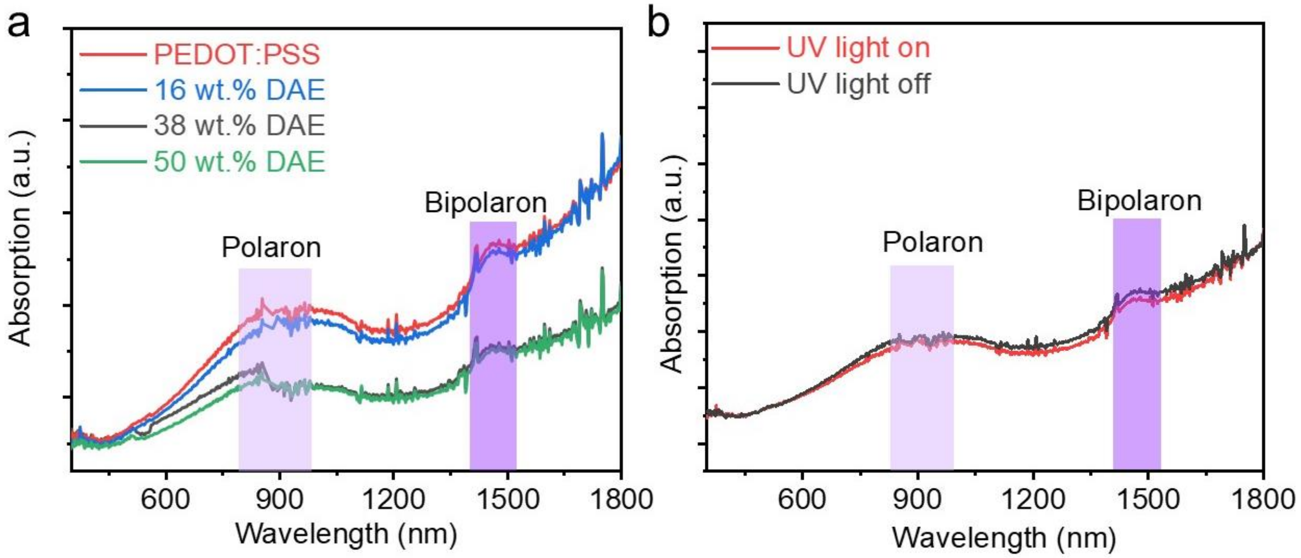

Fig. S20 (a) UV-visible-NIR absorption spectra of the PEDOT:PSS and PEDOT:PSS-*x*DAE (*x*=16, 38, 50 wt.%) films measured before and immediately after UV irradiation. **(b)** UV-visible-NIR absorption spectra of the PEDOT:PSS and PEDOT:PSS-*x*DAE (*x*=38 wt.%) films measured before and immediately after UV irradiation.

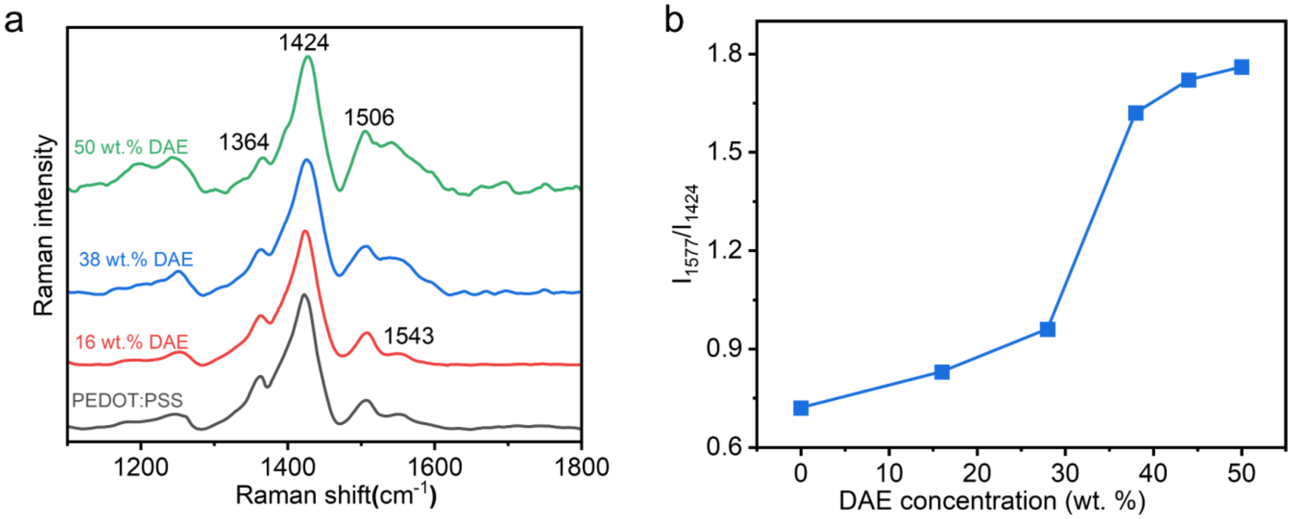

Fig. S21. (a) Raman spectra of the PEDOT:PSS and PEDOT:PSS-*x*DAE films under visible light. **(b)** Intensity ratios of the Raman peaks at 1577 cm^{-1} and 1424 cm^{-1} were measured under UV light for PEDOT:PSS-*x*DAE films made with different DAE concentrations.

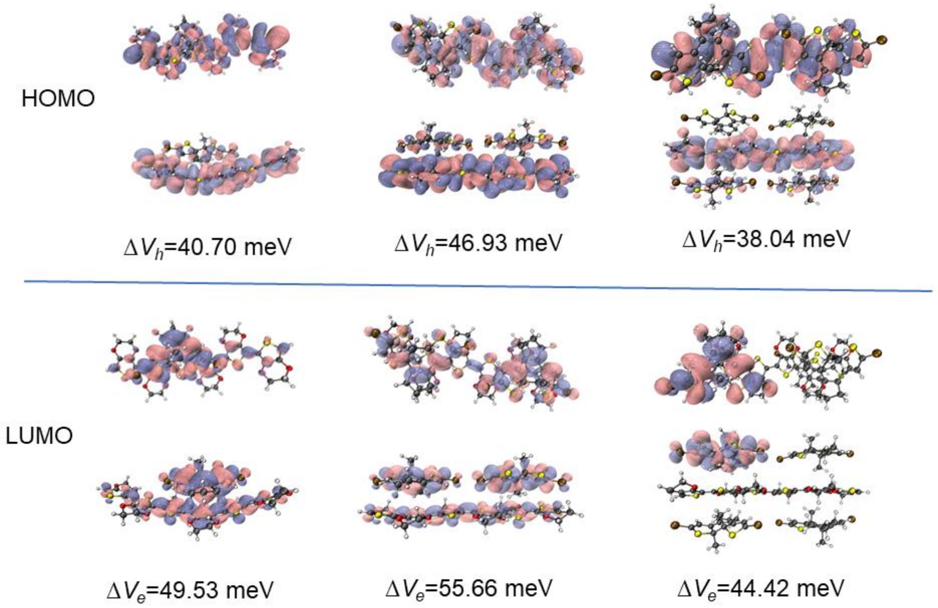

Fig. S22. Charge transfer integrals for 6EDOT coupled with one, two, and four DAE molecules.

Table S1. Gibbs coupling energy, HOMO and LUMO energies for coupling of one DAE molecule with six EDOT units (the configurations with degenerate states are marked with the red bold font).

	1DAE-1	1DAE-2	**1DAE-3**	1DAE-4
Gibbs coupling energy (meV)	-245.52	-340.28	**-341.39**	-324.88
HOMO (eV)	-4.03	-4.06	**-4.02**	-4.01
LUMO (eV)	-1.57	-1.59	**-1.58**	-1.54

Table S2. Gibbs coupling energy, HOMO and LUMO energies for coupling of two DAE molecules with six EDOT units (the configurations with degenerate states are marked with the red bold font).

	**2DAE-1**	**2DAE-2**	**2DAE-3**	2DAE-4	2DAE-5	2DAE-6	2DAE-7	2DAE-8
Gibbs coupling energy (meV)	**-404.54**	**-391.58**	**-394.77**	-258.75	-348.07	-323.67	-359.87	-321.88
HOMO (eV)	**-4.18**	**-4.21**	**-4.20**	-4.10	-4.11	-4.09	-4.17	-4.11
LUMO (eV)	**-1.79**	**-1.71**	**-1.73**	-1.68	-1.77	-1.70	-1.72	-1.72

Table S3. Gibbs coupling energy, HOMO and LUMO energies for three DAE molecules coupled with six EDOT units (the configurations with degenerate states are marked with the red bold font).

	**3DAE-1**	**3DAE-2**	**3DAE-3**	3DAE-4	3DAE-5	3DAE-6	3DAE-7	3DAE-8	3DAE-9
Gibbs coupling energy (meV)	**-417.11**	**-402.70**	**-400.55**	-420.82	-391.67	-395.02	-348.85	-370.05	-386.22
HOMO (eV)	**-4.25**	**-4.27**	**-4.26**	-4.37	-4.26	-4.26	-4.28	-4.32	-4.27
LUMO (eV)	**-1.81**	**-1.83**	**-1.82**	-1.83	-1.80	-1.82	-1.82	-1.81	-1.84

Table S4. Gibbs coupling energy, HOMO and LUMO energies for four DAE molecules coupled with six EDOT units (the configurations with degenerate states are marked with the red bold font).

	**4DAE-1**	4DAE-2	4DAE-3	4DAE-4	4DAE-5	**4DAE-6**	4DAE-7
Gibbs coupling energy (meV)	**-404.52**	-351.07	-355.82	-369.91	-355.87	**-391.17**	-372.99
HOMO (eV)	**-4.31**	-4.22	-4.45	-4.42	-4.30	**-4.29**	-4.28
LUMO (eV)	**-1.87**	-1.80	-1.92	-1.90	-1.89	**-1.90**	-1.85

Table S5. Thermopowers of the PEDOT:PSS-*x*DAE (*x*=38 wt.%) and PEDOT:PSS materials with different UV laser powers.

UV Laser Power (W)	0	0.35	1.08	2.09	3.31	4.58	5.74	6.95	8.11	9.24
Thermopower ($\mu V\ K^{-1}$) PEDOT: PSS-*x*DAE (*x*=38 wt.%)	13.01 ±0.57	17.54 ±1.54	21.66 ±1.35	40.32 ±2.35	62.18 ±2.71	80.08 ±4.77	92.68 ±5.23	113.55 ±7.84	131.91 ±5.94	132.04 ±8.29
Thermopower ($\mu V\ K^{-1}$) PEDOT: PSS	12.55 ±0.48	14.86 ±0.66	14.57 ±1.93	14.77 ±0.88	14.32 ±0.98	14.02 ±1.14	14.91 ±0.66	14.37 ±1.16	17.84 ±0.12	18.91 ±1.32

Table S6. Thermopowers of the PEDOT:PSS-*x*DAE (*x*=38 wt.%) and PEDOT:PSS materials at different temperatures.

Temperature (^{o}C)	10	20	30	40	50	60	70
Thermopower ($\mu V\ K^{-1}$) PEDOT: PSS-*x*DAE (*x*=38 wt.%)	69.74 ±1.87	63.09 ±2.18	62.63 ±1.40	57.91 ±3.12	51.03 ±2.63	43.49 ±3.28	36.67 ±1.4
Thermopower ($\mu V\ K^{-1}$) PEDOT: PSS	11.34 ±0.97	11.72 ±0.82	11.85 ±0.49	12.51 ±0.60	12.81 ±0.93	13.30 ±0.97	16.45 ±0.52

Table S7. Thermopowers of the PEDOT:PSS-*x*DAE (*x*=38 wt.%) and PEDOT:PSS materials with different exposure area percentages.

Exposure area percentage (%)	20	40	60	80	100
Thermopower (μV K^{-1}) PEDOT: PSS-*x*DAE	33.38 ±2.74	41.73 ±2.95	54.83 ±2.72	63.30 ±2.87	65.94 ±3.33
Thermopower (μV K^{-1}) PEDOT: PSS	13.23 ±0.74	12.68 ±0.71	13.06 ±0.91	12.36 ±1.08	13.37 ±1.26

Table S8. Thermopowers of the PEDOT:PSS-*x*DAE (*x*=38 wt.%) and PEDOT:PSS materials with different angles of light incidence.

Angle of light incidence (^{o})	70	60	50	40	30	20	10	0
Thermopower (μV K^{-1}) PEDOT: PSS-*x*DAE	24.02 ±2.42	31.27 ±2.97	40.67 ±2.91	55.28 ±3.86	59.77 ±5.34	64.99 ±3.61	67.59 ±3.03	69.07 ±4.03
Thermopower (μV K^{-1}) PEDOT: PSS	12.18 ±0.63	12.80 ±0.93	12.67 ±0.54	12.93 ±0.85	14.61 ±0.66	14.90 ±0.91	15.52 ±0.87	15.05 ±0.98

Table S9. Thermopowers of the PEDOT:PSS-*x*DAE materials prepared with different DAE concentrations with the UV light on (UV power density was 1.3 W cm^{-2}) and off.

DAE concentration (wt. %)	0	16	28	38	44	50
thermopower (μV K^{-1}) with the UV light on	19.35 ±3.08	61.69 ±3.73	90.80 ±2.88	135.46 ±4.9	120.33 ±3.50	114.92 ±3.37
thermopower (μV K^{-1}) with the UV light off	12.37 ±0.96	12.96 ±0.78	13.51 ±0.87	13.58 ±0.54	17.29 ±0.39	17.85 ±1.25

Table S10. Thermopowers of the PEDOT:PSS-*x*DAE (*x*=38 wt.%) and PEDOT:PSS materials doped with the ionic liquid Li:TFSI and dedoped with LAA with the UV light on (the UV power density was 1.3 W cm^{-2}) and off.

	20 wt.% Li:TFSI	40 wt.% Li:TFSI	PEDOT: PSS	20 wt.% LAA	40 wt.% LAA
Thermopower (μV K^{-1}) with the UV light on	108.04 ±4.22	104.04 ±3.83	131.38 ±4.55	94.04 ±3.49	83.17 ±3.38
Thermopower (μV K^{-1}) with the UV light off	11.37 ±1.27	9.90 ±0.83	12.98 ±0.86	19.22 ±1.16	23.40 ±1.19
